# Numerical modeling of internal tides and submesoscale turbulence in the US Caribbean regional ocean

**DOI:** 10.1038/s41598-023-27944-2

**Published:** 2023-01-19

**Authors:** Sonaljit Mukherjee, Doug Wilson, Paul Jobsis, Sennai Habtes

**Affiliations:** 1 Center for Marine and Environmental Studies, University of the Virgin Islands, 2 John Brewers Bay, Charlotte Amalie, VI 00802 USA; 2 USVI Department of Planning and Natural Resources, 84P5+PX3, Anna’s Retreat, Charlotte Amalie, VI 00802 USA

**Keywords:** Ocean sciences, Physical oceanography

## Abstract

The US Caribbean ocean circulation is governed by an influx of Atlantic water through the passages between Puerto Rico, Hispaniola and the Virgin Islands, and an interplay of the Caribbean Sea water with the local topography of the region. We present an analysis of the US Caribbean ocean flow simulated by the USCROMS; which is the ROMS AGRIF model configured for the US Caribbean regional ocean at a horizontal resolution of 2 km. Outputs from the USCROMS show a seasonal variability in the strength of submesoscale turbulence within a mixed layer whose depth varies from −70 to −20 m from winter to summer, and internal tides originating from the passages between the islands. Energy spectra of the simulated baroclinic velocity show diurnal and semi-diurnal maxima and several higher-order harmonic frequency maxima associated with non-linear internal waves forming over steep slopes with super-critical topography in the continental shelf. The strongest conversion rates of the depth-averaged barotropic to baroclinic tidal energy occur at localized regions in the continental shelf with super-critical topography. These regions also exhibit enhanced transport and dissipation of the depth-averaged barotropic and baroclinic tidal kinetic energy. The dissipation in these regions is nearly 3 orders of magnitude stronger than the open ocean dissipation. The energy transport terms show a seasonal pattern characterized by stronger variance during summer and reduced variance during the winter. At the benthic regions, the dissipation levels depend on the topographic depth and the tidal steepness parameter. If the benthic region lies within the upper-ocean mixed-layer, the benthic dissipation is enhanced by surface-forced processes like wind forcing, convective mixing, submesoscale turbulence and bottom friction. If the benthic region lies below the mixed-layer, the benthic dissipation is enhanced by the friction between the super-critical topographic slopes and the periodically oscillating baroclinic tidal currents. Due to bottom friction, the tidal oscillation in the lateral currents adjacent to the sloping topography generates cyclonic and anti-cyclonic vortices with O(1) Rossby number depending on the orientation of the flow. While the cyclonic vortices form positive potential vorticity (*q*) leading to barotropic shear instability, anti-cyclonic vortices form negative *q* which leads to periodically occurring inertial instability. The lateral and inertial instabilities caused by the baroclinic tidal oscillations act as routes to submesoscale turbulence at the benthic depths of −100 m to −400 m near the super-critical topography of the continental shelf, forming O(10 km) long streaks of turbulent water with dissipation levels that are 3 orders of magnitude stronger than the dissipation in the open ocean at the same depth. The magnitudes of the dissipation and *q* at the benthic regions over super-critical continental-shelf topography are also modulated by the spring-neap tidal signals.

## Introduction

**Figure 1:**
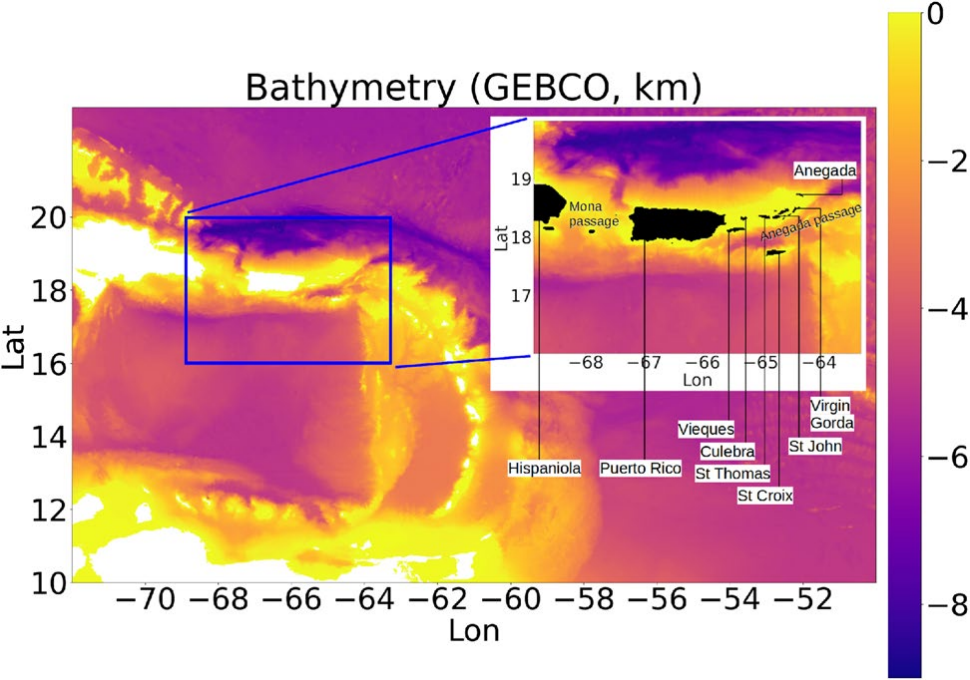
Map showing the Caribbean sea topography from the General Bathymetric Chart of the Oceans (GEBCO). Blue rectangle is the domain covered by USCROMS parent grid. The islands St Thomas, St Croix and St John form the US Virgin Islands (USVI). The islands Anegada and Virgin Gorda form the British Virgin Islands (BVI).

The US Caribbean ocean comprises of the local ocean surrounding Puerto Rico and the Virgin Islands (Fig. 1). These islands are part of the Antilles island belt along the northern and eastern boundaries of the Caribbean Sea. The ocean circulation in the Caribbean Sea is characterized by an influx of Atlantic ocean water through the passages between the Antilles islands, flowing towards the Gulf of Mexico. The passages between Puerto Rico and the US Virgin Islands, the Mona passage between Puerto Rico and Hispaniola, and the Anegada passage to the east of Anegada (British Virgin Islands), serve as conduits for the inflow of Atlantic ocean water into the Caribbean sea.

Coastal ocean circulation around the islands is influenced by a number of factors: complex topographic features around the islands, tidal transport, and seasonality in the atmospheric forcing. The interaction of open-ocean eddies and complex coastal topography generates instabilities that lead to submesoscale turbulence in the upper ocean and near the benthic depths. For example, the sloping topography of the Florida shelf had been shown to generate anti-cyclonic vortices while interacting with the Florida current, leading to topographically generated symmetric instability, turbulence and dissipation^1^. Idealized numerical simulations of the flow around a simulated island have shown the emergence of continuous cyclonic and anti-cyclonic vortices from the edges of the island mass^2^. Seamounts in the Tokara strait, south of Kyushu Japan, generate continuous streaks of negative potential vorticity leading to an increase in the turbulence and dissipation by 3 orders of magnitude over a depth range of −100 m to −300 m below the surface^3^. These aforementioned studies have focused on the enhancement of benthic turbulence by instabilities created by the mesoscale and submesoscale currents over the continental shelf. However, internal tides can also play an important role in the formation of benthic turbulence over the continental shelf topography^4–6^. Depending on the topographic characteristics and the horizontal distance of the location from the islands, the intensity of tidal oscillations may vary. It is therefore important to identify the routes to the formation of benthic turbulence by tidal oscillations, and explore the spatial and temporal variability in the intensity of tidally forced turbulence in the benthic waters.

The generation of internal tides over complex topographic features have been assessed in various observations and studied using numerical modeling. For example, measurements from autonomous gliders deployed in the Luzon Strait have shown the propagation of internal tides^7,8^. The depth-integrated conversion of barotropic to internal tidal energy has been derived using prognostic equations of the fluid density and momentum, and studied using numerical simulations with realistic coastal topography^9,10^. Strong magnitudes of the depth-integrated conversion rate of the barotropic to baroclinic tidal energy have been found to occur in the coastal regions with steep topographic slopes in the continental shelf^10–12^. Regions with sub-critical topography ($\phi < 1$, $\phi$ is the steepness parameter defined in Online Appendix 6) generate mostly first mode internal tides at the forcing frequency of the barotropic tide, whereas higher-mode internal tides are generated at harmonic frequencies by super-critical topography ($\phi > 1$). Depending on the barotropic tidal velocity amplitude, internal tides originating in the regions with large excursion lengths have more energy content in the higher harmonic modes than the first mode^13^. Tall and steep topographic structures with tidal excursion parameters less than 1 ($\psi < 1$, $\psi$ is the excursion parameter, Online Appendix 6) have been shown to generate unsteady Lee waves associated with high turbulent dissipation^14,15^. Low-mode internal tides transport larger tidal energy and propagate over long distances, while the high-mode internal tides dissipate locally near the source zones^16–18^.

The intensity of internal tidal energy and transport have been shown in both observations and numerical simulations to respond to the seasonal and spring-neap variations in the upper ocean properties. For example, measurement of nutrient fluxes in the shelf edge of the Celtic Sea has shown that spring tides resulted in higher nutrient fluxes due to enhanced turbulence and dissipation^19^. Numerical simulations of semi-diurnal internal tides in the east Japan sea^20^ have shown that the depth-integrated internal tidal energy in the coastal regions have a seasonal pattern characterized by higher energy levels during the summer months and lower energy during the winter months. Hydrostatic simulations in the Bay of Biscay have shown that the seasonal changes in the thermocline depth have an impact on the intensity of the depth-integrated conversion rate of surface to internal tidal energy^21^. The contrast in the strength of internal tidal energy and conversion rates between the winter and summer months is attributed to the seasonal changes in the upper ocean stratification. Winter months are characterized by deeper mixed-layer (*ML*) and enhanced upper-ocean submesoscale turbulence which strengthens the mixing and dissipation within the *ML* compared to the summer months^22^. Therefore, the depth-averaged conversion rates in the coastal regions with shallow topography are likely to exhibit a stronger response to the seasonal deepening and shallowing of the *ML* compared to the open-ocean regions with topographic depths larger than O(1 km). It is also important to find out what is the extent of depth to which the seasonal and spring-neap characteristics in the tidal oscillations are influential to the formation of turbulence and dissipation.

The US Caribbean ocean boasts a vibrant marine ecosystem monitored by the marine researchers at the University of the Virgin Islands (UVI). Understanding the physical characteristics of the ocean around the islands helps the researchers to better assess the impact of the physical processes on the coral reef resilience and larval spawning and transport. However, due to sparsely available ocean data, the physical oceanographic characteristics of this region have not been explored in detail. Glider measurements and ADCP data near the coast of the Virgin Islands have shown the presence of semi-diurnal and diurnal tidal signals. The coastal topography around the US Caribbean islands is highly complex with super-critical steepness at many locations which are likely to generate strong internal tidal oscillations. Therefore, a detailed analysis on the spatial, temporal and seasonal variability of the tidal kinetic energy transport and dissipation in the US Caribbean region is necessary to estimate the impact of the tidal motions on the marine ecosystems.

In this work, we attempt to understand the physical characteristics of the US Caribbean regional ocean using numerical modeling. We focus our studies on the seasonality of submesoscale turbulence, and the generation, transport and dissipation of internal tides. We explore the spatial variability of the barotropic and baroclinic tidal kinetic energy transport depending on the topographic characteristics, and the response of the kinetic energy transport to the seasonal changes in stratification and the spring-neap oscillations. We also explore the routes to the formation of benthic turbulence by tidal oscillations, the dependence of benthic turbulent dissipation on the topographic characteristics, and the impact of seasonal and spring-neap tidal variability in the water column on the turbulence and dissipation.

This paper is outlined as follows. Section “USCROMS” discusses the numerical modeling system used in our research. In the “Results” section, we discuss our analysis on the model outputs: seasonality in submesoscale turbulence, spatio-temporal and seasonal variability in the generation and transport of internal tidal kinetic energy, and the impact of internal tidal oscillations on the benthic turbulence and dissipation. Section “Conclusion” concludes our research.

## USCROMS

The US Caribbean Regional Ocean Modeling System (USCROMS) is a split-explicit primitive equation ocean modeling system based on the ROMS AGRIF (Regional Ocean Modeling System with Adaptive Grid Refinement in Fortran) architecture^23,24^. It simulates the ocean properties by numerically solving the geophysical Navier-Stokes equations in a 3-dimensional grid that is orthogonal-curvilinear along the horizontal, and has a terrain-following $\sigma$ coordinate system along the vertical^24^. The depth of the vertical layers (called $\sigma$ layer) of the USCROMS grid varies with the topography, and the top face of the top-most grid is the sea surface.

The USCROMS is composed of a 3-dimensional grid with a 2 km horizontal resolution. The grid uses climatological data on the ocean properties from the Mercator Daily Global Physical Bulletin at $1/12^0$ (PSY4)^25^. The vertical coordinate system is designed as a continuous double-stretching function of the bottom topography with a total of 32 grid levels. The bottom topography is smoothed using a 2-dimensional Gaussian filter, achieving a Haidvogel number of 0.17 and a subsequent Haney number of 3.7. The model parameters are given in Table 1.

**Figure 2:**
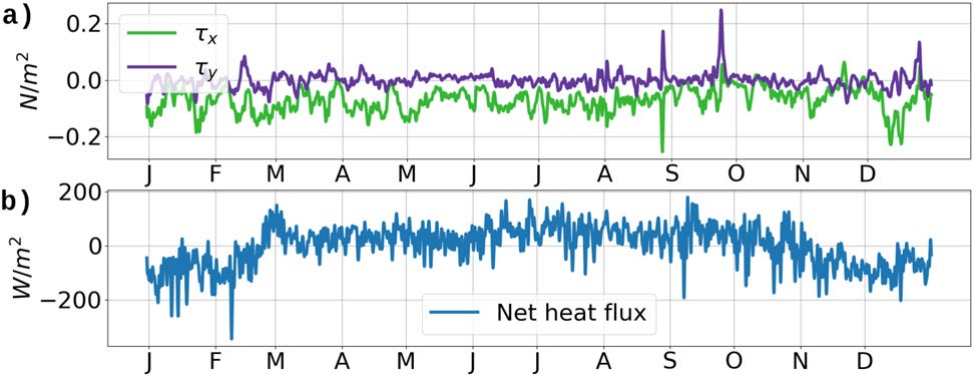
(**a**) 6-hourly averaged wind stress from CERSAT in 2019. (**b**) Hourly net heat flux from the Atlantic PIRATA moored buoy in 2019. The data plotted here are lowpassed with a $1/24~\mathrm{hr}^{-1}$ cutoff for display. Original data was used in the simulation

For atmospheric forcing, the USCROMS uses the 6-hourly wind-stress data from IFREMER CERSAT Global Blended Mean Wind Fields^26^, and hourly heat flux data from the PIRATA Atlantic mooring station. Time series data of the wind stress and the heat flux used in our simulation is shown in Fig. 2a,b. Tidal forcing is obtained from the TPXO tides package^27^. Using the TPXO package, the simulated sea-surface height from USCROMS is strongly correlated with tidal-gauge water levels measured near St Thomas, Vieques and Puerto Rico, with coefficients close to 0.9.

We initialized the USCROMS with climatology data from the 2018 Mercator Daily Global Bulletin dataset at $1/12^0$ (resolution 10 km), and ran a 1-year long simulation using the 2018 surface forcing data to reach a quasi-steady spin-up state. Then, we used the final outputs from the 2018 simulation to initialize the model for the 2019 simulation. The Daily Global Bulletin data is provided at the USCROMS boundary walls every 5 days, and the AVISO sea-surface altimetry data is provided to USCROMS at every day.

The purpose of eddy viscosity and diffusivity is to maintain a simplistic parameterization of the rate of turbulent kinetic energy transfer from the resolved scales to the subgrid scales unknown to the model due to the limitation imposed by the grid resolution. Therefore, the dissipation, parameterized as the product of the eddy viscosity and the shear-squared, serves as a rate of transfer of the turbulent eddy kinetic energy from the resolved scales to the subgrid scales^28,29^.

**Table 1:** USCROMS parameters

Horizontal resolution and mode	2 *km*, hydrostatic
Vertical grid	32 levels, terrain-following $\sigma$ coordinates
Horizontal grid	Orthogonal curvilinear
Model topography	GEBCO^30^, smoothed using 2D Gaussian filter
	Haidvogel number 0.17, Haney number 3.7
Horizontal advection/diffusion	5th order WENO^31^
	Smagorinsky^32^ mixing
Vertical advection/diffusion	5th order WENO
	K-profile parameterization^33^
Boundary condition	Radiative boundary condition^34^
	6 *km* sponge layer with nudging timescale of 1 day
Climatology	Mercator Daily Physical Bulletin $1/12^0$
Sea-surface height	AVISO Altimetry
Heat flux and wind stress	PIRATA Atlantic mooring data (hourly)
	IFREMER CERSAT^26^ (6 hourly)

## Results

### Mixed-layer depth

The mixed-layer depth (*MLD*) in our analysis is calculated as a depth where the water density is $0.03~{ \mathrm {kg}}/\mathrm{{m}}^3$ higher than the density at a reference depth of −10 m from the surface^35^.

**Figure 3:**
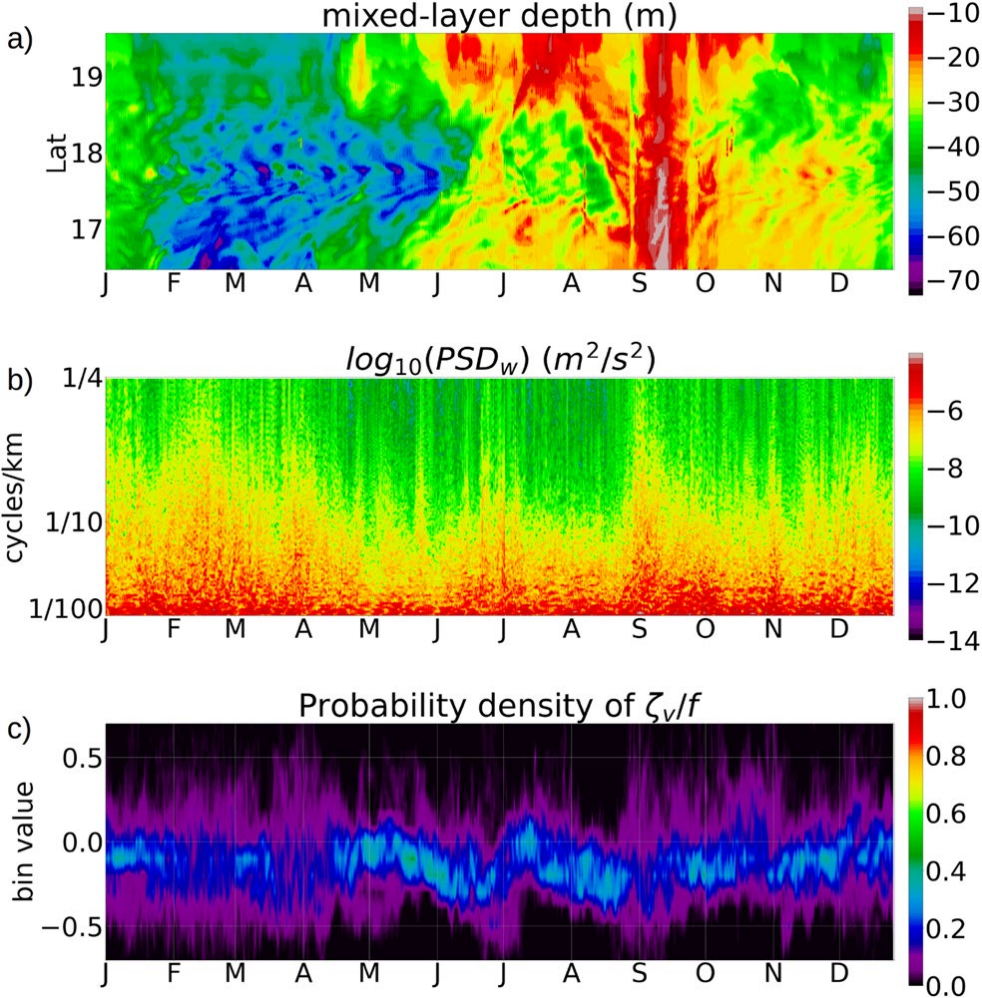
(**a**) Hovmöller diagram of the *MLD* along a north-south transect passing through longitude $-65.1^0~W$, between St Thomas and Culebra. (**b**) Power spectral density (PSD) of the de-tided vertical velocity at −7 m depth along the north-south transect passing through longitude $-65.1^0~W$. (**c**) Probability density of the normalized vertical relative vorticity $\zeta _v/f$, computed from the de-tided current velocities at −7 m depth, along the same transect. The y axis shows the minimum value of each bin, and the bin size is 0.1.

The upper-ocean *ML* in the Caribbean Sea deepens to −70 m during the winter months from January to April, and shallows to −20 m during the subsequent summer months (Fig. 3a).

The spatio-temporal scales corresponding to the fastest growing mode of ageostrophic baroclinic instability (*ABI*) are expressed as $L_{ABI} = \frac{2\pi U }{\vert f \vert } \sqrt{\frac{1 + Ri}{5/2}}$ and $T_{ABI} = \sqrt{\frac{54}{5}}\sqrt{\frac{1+Ri}{\vert f \vert }}$^36,37^ where *U* is the geostrophic velocity scale, $Ri = N^2 M^4/f^2$ is the geostrophic Richardson number within the mixed layer ($N^2$ and $M^2$ are the vertical and lateral buoyancy frequencies respectively), and *f* is the Coriolis frequency. The typical values of the geostrophic velocity scale *U* obtained from our simulations is 0.08 *m*/*s*. Using *U*, we obtain a spatial scale $L_{ABI} = 7.5~\mathrm{{km}}$ and a temporal scale $T_{ABI}=21~hr$ for the fastest growing *ABI* mode.

### Submesoscale seasonality

The variability in the strength of submesoscale turbulence within the *ML* had been quantified in several observations^38^ and demonstrated in numerical simulations^22,39–41^. Deeper *ML* results in a stronger conversion of available potential energy (*APE*) to submesoscale eddy kinetic energy through mixed-layer instabilities, and subsequently, stronger fluctuations in the vertical velocity. Since submesoscales are characterized by O(1) Rossby numbers ($\vert \zeta _{v}/f \vert$, where $\zeta _{v}$ is the relative vertical vorticity, *f* is the Coriolis frequency) and O(1) Richardson numbers^42^, the probability of occurrence of O(1) magnitudes of $\vert \zeta _v/f \vert$ is higher during winter when the mixed-layer is deeper, compared to the summer season^40,41^.

Hovmöller diagram of the *MLD* from the USCROMS outputs along a north-south transect passing through the west of St Thomas, shows that the *ML* deepens to −70 m during the winter months of January to May, followed by shallowing of the *ML* to −20 m from June to October, and subsequent deepening during November and December (Fig. 3a). The power spectral density (PSD) of the vertical velocity *w* (Fig. 3b) shows strong spectral amplitudes during the winter months of January to April, followed by weakened amplitudes from May to August. During August and September, a strong spike in the wind stress (Fig. 2) results in the *ML* deepening to −40 m at the south side of St Croix, along with stronger spectral amplitudes of *w*. A probability density function (*PDF*) of the normalized relative vorticity $\zeta _v/f$ (Fig. 3c) shows that during the winter months of January to April, $\zeta _v/f$ appears to be more diffused and spread away from the 0 axis, indicating a higher number of locations with $\vert \zeta _v/f \vert$ near 0.5. During May and June, the PDF is mostly concentrated near the 0.0 axis, indicating that $\vert \zeta _v/f \vert$ is close to 0 which happens during reduced submesoscale activity. The regions where the PSD is spread out towards the higher values, coincide with stronger spectral amplitudes in the vertical velocity. During the month of September, a spike in the wind stress results in a deeper *ML*, causing an increase in the spectral amplitudes of *w* and a more diffused *PDF* of $\zeta _v/f$.

### Internal tides

**Figure 4:**
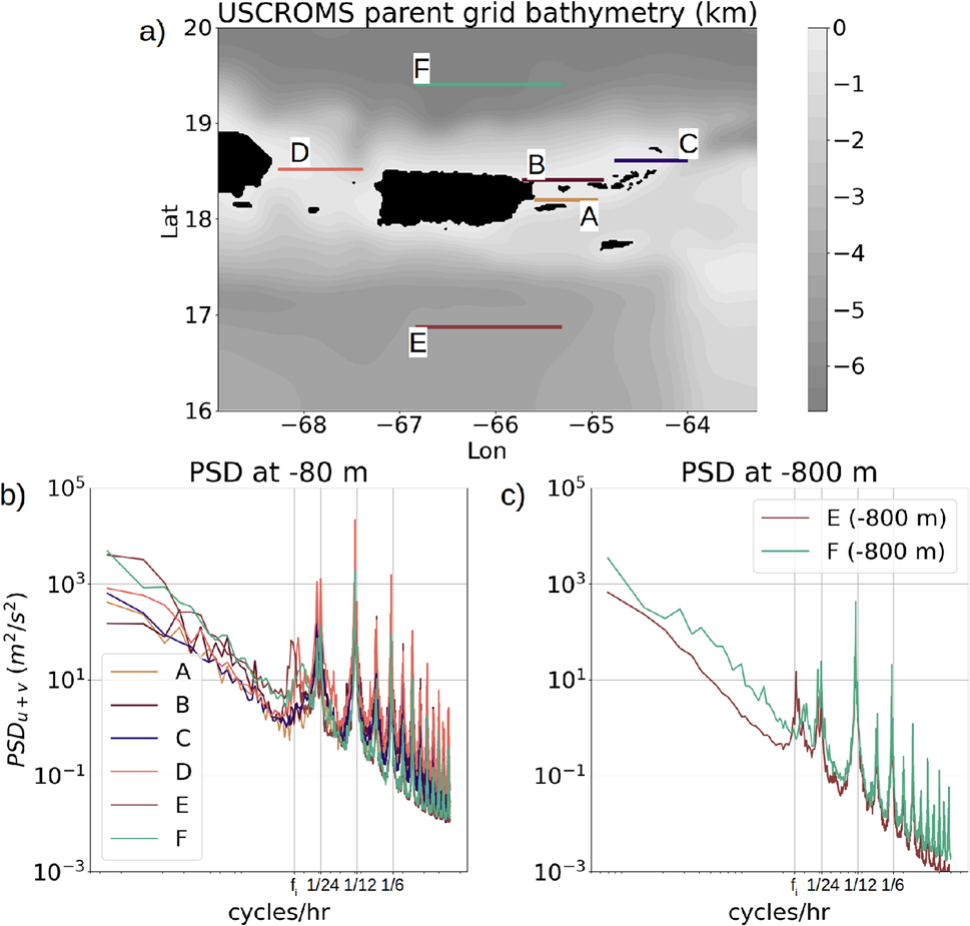
(**a**) USCROMS parent grid with 6 lateral transects marked in colors. For each transect, the power spectral density ($\mathrm {PSD}_{u+v}$) of the horizontal baroclinic velocity is calculated as an average of the PSD of the velocity time-series over 2 months (October to November) at each grid point along the transect. (**b**) Averaged PSD for all transects at −80 m depth. (**c**) Averaged PSD for the transects E and F at −800 m depth.

Baroclinic current velocity at a depth of −80 m and −800 m (Fig. 4a, b, c) shows several maxima in the spectral amplitude at different frequencies. The maxima at the frequencies $1/24~\mathrm{{hr}}^{-1}$ and $1/12~\mathrm{{hr}}^{-1}$ are due to the diurnal and semi-diurnal internal tides respectively. There are several spectral maxima at frequencies higher than the semi-diurnal frequency which are associated with higher-harmonic internal waves generated by non-linear interaction of the barotropic tides with the steep topographic slopes close to the islands^6,15,43^. An inertial peak is also noted in the baroclinic current PSD (Fig. 4b, c), which suggests the presence of near-inertial waves. The near-inertial peak at −80 m depth appears stronger in magnitude compared to the near-inertial peak at −800 m depth for the open-ocean transects E and F. It has been shown in numerical modeling and observational studies that near-inertial waves are generated through inertial oscillations caused by lee waves propagating over the bottom topographic features^3,44^. In the upper ocean, submesoscale frontal instabilities and surface wind forcing have also been noted to generate near-inertial waves^45^.

**Figure 5:**
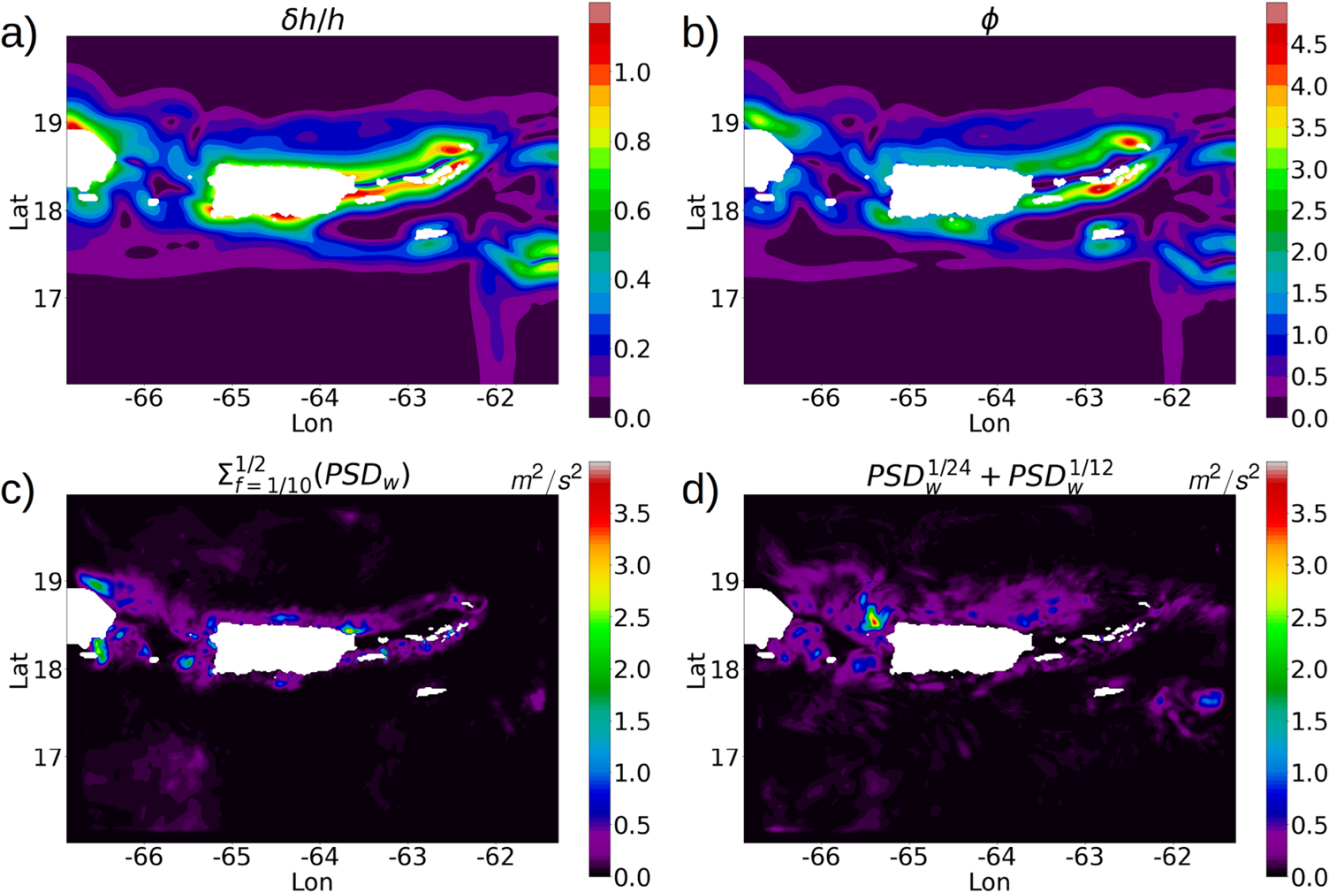
(**a**) Normalized topographic gradient $\delta h/h$. (**b**) Tidal steepness parameter (Online Appendix 6) for the semi-diurnal internal tide computed with $N^2$ values at the first $\sigma$ surface (figure S1, supplementary material). (**c**) Contours showing the summation of the vertical velocity (*w*) PSD values over a frequency range of $1/10~\mathrm{{hr}}^{-1}$ to the cut-off frequency $1/2~\mathrm{{hr}}^{-1}$ ($1/10~\mathrm{{hr}}^{-1}$ was chosen because it is slightly higher than the semi-diurnal frequency). (**d**) Summation of the PSD values of *w* for the frequencies $1/24~\mathrm{{hr}}^{-1}$ and $1/12~\mathrm{{hr}}^{-1}$. The PSD is computed at each location from a 2 month (October to November) time series of *w* over the 1st $\sigma$ surface.

The spatial distribution of the energy of the non-linear higher-harmonic internal waves is displayed in Fig. 5c showing the contours of $\Sigma _{f=1/10}^{1/2}PSD_w$ which is the sum of the vertical velocity spectral variances within a frequency range from $1/10~\mathrm{{hr}}^{-1}$ to the cut-off frequency $1/2~hr^{-1}$, computed on the first $\sigma$ surface of the vertical grid (height of the first $\sigma$ surface from the topographic depth is shown in figure S1, supplementary material). Strong energy levels for the higher-harmonic internal waves occur in the regions close to the islands with super-critical topography (tidal steepness value $\phi > 1.0$) and $\delta h/h$ values from 0.4 to 1.0: the western edge of Anegada, southern coastline of St Thomas, the region between Vieques and Culebra, and localized regions along the coastline of Hispaniola and western Puerto Rico (Fig. 5a, b). The spatial distribution of the combined energy of the diurnal and semi-diurnal internal waves ($PSD_w^{1/24} + PSD_w^{1/12}$) is similar to the higher-harmonic internal wave energy distribution but more spread out to the northern open ocean, especially around the northern coastline of Puerto Rico (Fig. 5d). Internal waves also originate in the southwest part of the domain in the Caribbean sea where the depth is typically close to 4 *km* and the steepness parameter is close to 0. The northern coastlines of St Thomas, St John, BVI (excluding Anegada) and Vieques, where the topography is relatively shallow and flat with steepness parameters less than 1.0, shows very weak internal-wave energy.

The transects A, B and C at the passages between the islands show stronger spectral amplitudes than the transects D, E and F in the open ocean (Fig. 4b), which is a consequence of the locations being close to the island landmasses characterized by shallow depths and stronger production of the internal tidal kinetic energy (discussed in the “Kinetic energy analysis” section). Numerical simulations of internal tides^10^ have shown that the generation of internal tidal kinetic energy is higher in the regions close to the landmass where the topography is shallow. Low-mode internal tides originating from shallow coastal regions propagate over long distances of O($10^3~\mathrm{{km}}$), while tides generated at higher modes get mixed and dissipated rapidly^16,18^.

The spatial distribution of the internal wave energy at higher-harmonic tidal frequencies motivates further investigation on the rate of conversion of the barotropic to baroclinic tidal energy. In the next section, we look into the production of baroclinic tidal energy from barotropic tides.

### Conversion of barotropic to baroclinic energy

Baroclinic tidal waves originate over the topographic surface when the barotropic oscillating tides collide with the topographic slopes. The tidal steepness parameter of the topography determines the intensity of energy transferred from the barotropic to internal tides^10^. The expression for the rate of conversion of energy (referred to as *C*) from the barotropic to baroclinic energy is given in Online Appendix 9. The term *C* acts as a source to the depth-averaged net baroclinic energy which is the summation of the baroclinic kinetic energy ($E_{BCK}$) and available potential energy by interior density perturbations^9,12,46^. Likewise, $-C$ acts as a sink to the net barotropic energy which is the summation of the barotropic kinetic energy ($E_{BTK}$) and the barotropic perturbation potential energy.

**Figure 6:**
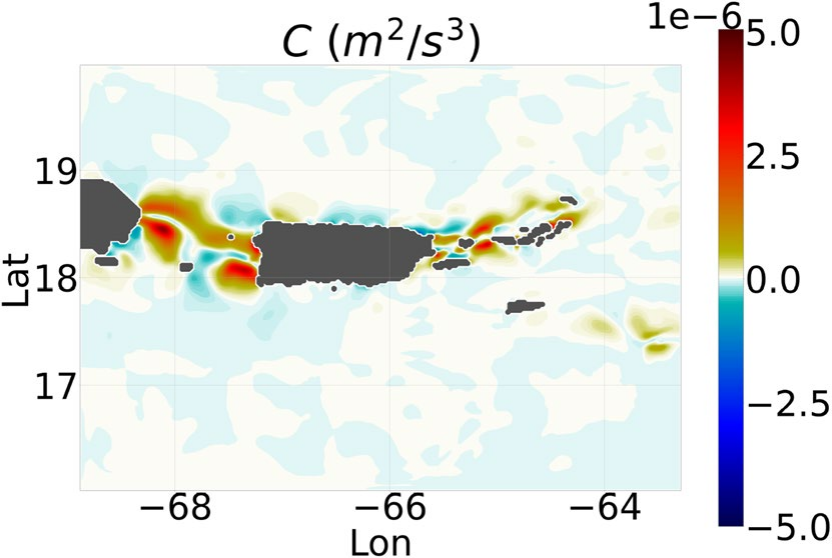
Depth-averaged conversion rate *C* of barotropic to baroclinic energy, averaged over 1 month in April. The averaging over 1 month is done to minimize the mesoscale and submesoscale perturbations. Details on the term *C* are provided in Online Appendix 9.

**Figure 7:**
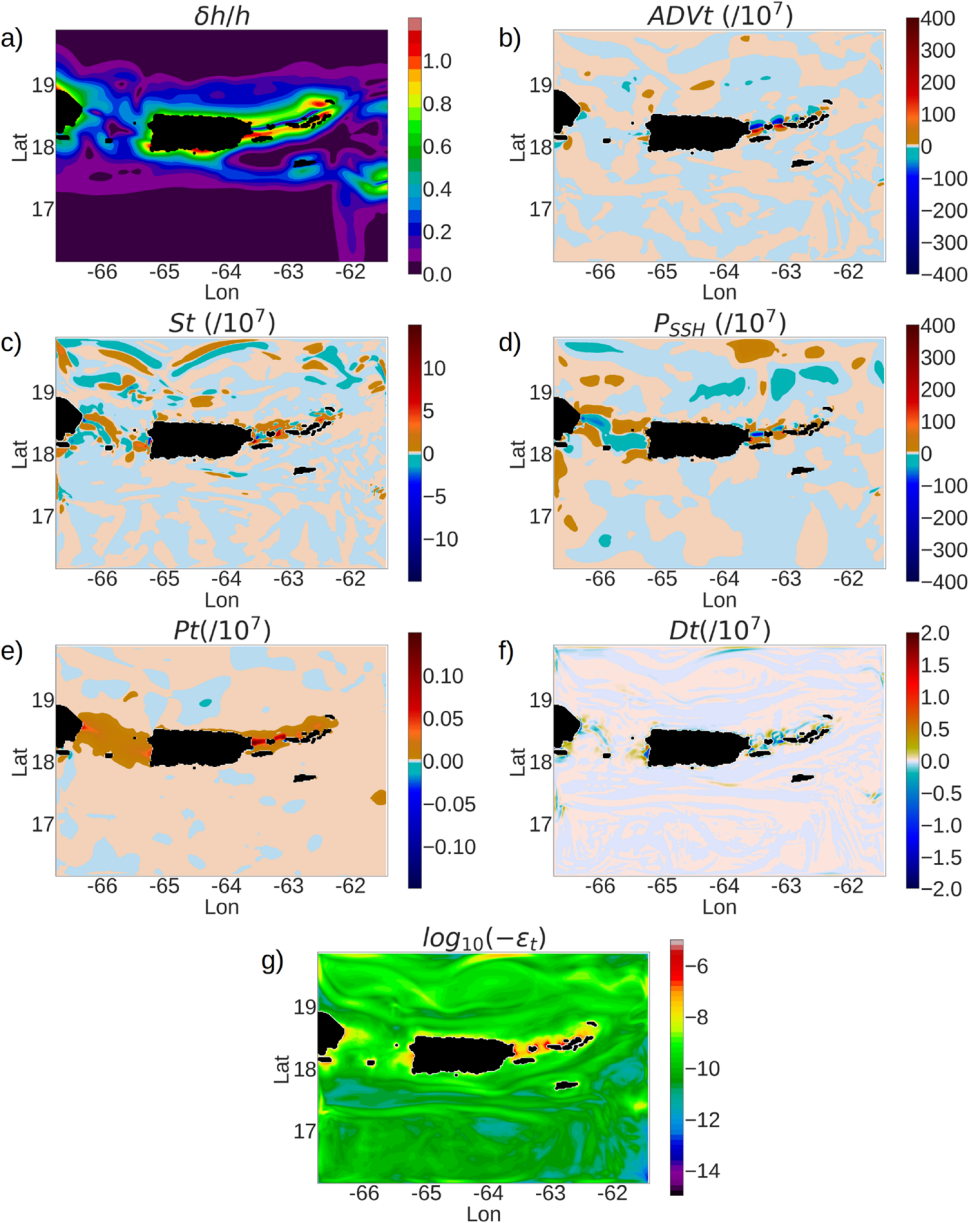
Snapshots of the different terms in the depth-averaged $E_{BTK}$ prognostic Eq. B8 (Online Appendix 7). (**a**) $\delta h/h$, (**b**) *ADVt*, (**c**) *St*, (**d**) $P_{SSH}$, (**e**) *Pt*, (**f**) *Dt*, (**g**) $\epsilon _t$. All components are averaged over 1 month in order to minimize the mesoscale and submesoscale perturbations. The color ranges are different for the individual plots in order to show the flow patterns prominently.

When averaged over 1 month, a contour plot of the depth-averaged conversion rate *C* (Fig. 6) shows that the highest rates of conversion of barotropic to baroclinic tidal energy occur in the passages between the islands: the Mona passage between Puerto Rico and Hispaniola, the passages between Saint Thomas and Culebra, Vieques and Puerto Rico, and the passage between Anegada and BVI. These regions with high magnitudes of conversion rate are characterized by normalized topographic-gradient ($\delta h/h$) values ranging from 0.4 to 1.0 (Fig. 7a), and super-critical topography with steepness parameter values greater than 1.0 (Fig. 5a).

### Kinetic energy analysis

To study the spatio-temporal variability of the kinetic energy production and transport associated with tidal motions, we examine the individual components of the depth-averaged prognostic equations of the barotropic ($E_{BTK}$) and the baroclinic ($E_{BCK}$) kinetic energy (see Online Appendix 7). All components of the kinetic energy prognostic equations are averaged over 1 month such that the mesoscale and submesoscale characteristics are minimized, whereas the tidal characteristics are retained since they are periodic in nature.

#### Barotropic kinetic energy

In a hydrostatic setup, the components of the depth-averaged $E_{BTK}$ are the advective transport (*ADVt*), baroclinic stress divergence (*St*), barotropic pressure work ($P_{SSH}$), baroclinic pressure work (*Pt*), diffusion (*Dt*) and dissipation ($\epsilon _t$) (refer to Eq. (B8) in Online Appendix 7). We study the spatial variability of the terms of the depth-averaged $E_{BTK}$ transport equation with respect to the normalized topographic gradient $\delta h/h$. Since $\delta h/h$ is inversely proportional to the depth *h* and directly proportional to the gradient $\delta h$, it holds high values of O(1) at the regions with steep topographic slopes and shallow depths.

Contour plots showing the spatial distribution of the components of $E_{BTK}$ equation show that *ADVt* and $P_{SSH}$ acquire magnitudes at O($10^{-5}~m^2/s^3$) in the passages between St Thomas, Culebra and Puerto Rico, nearly 3 orders of magnitude stronger than the baroclinic pressure work *Pt* (Fig. 7b, d, e). The baroclinic stress divergence term *St* holds magnitudes of O($10^{-6}~m^2/s^3$) in the same regions (Fig. 7c), an order of magnitude stronger than *Pt*. The depth-averaged diffusion and dissipation in these regions are at O($10^{-6}~m^2/s^3$), nearly 3 times stronger than the open ocean diffusion and dissipation (Fig. 7f, g).

**Figure 8:**
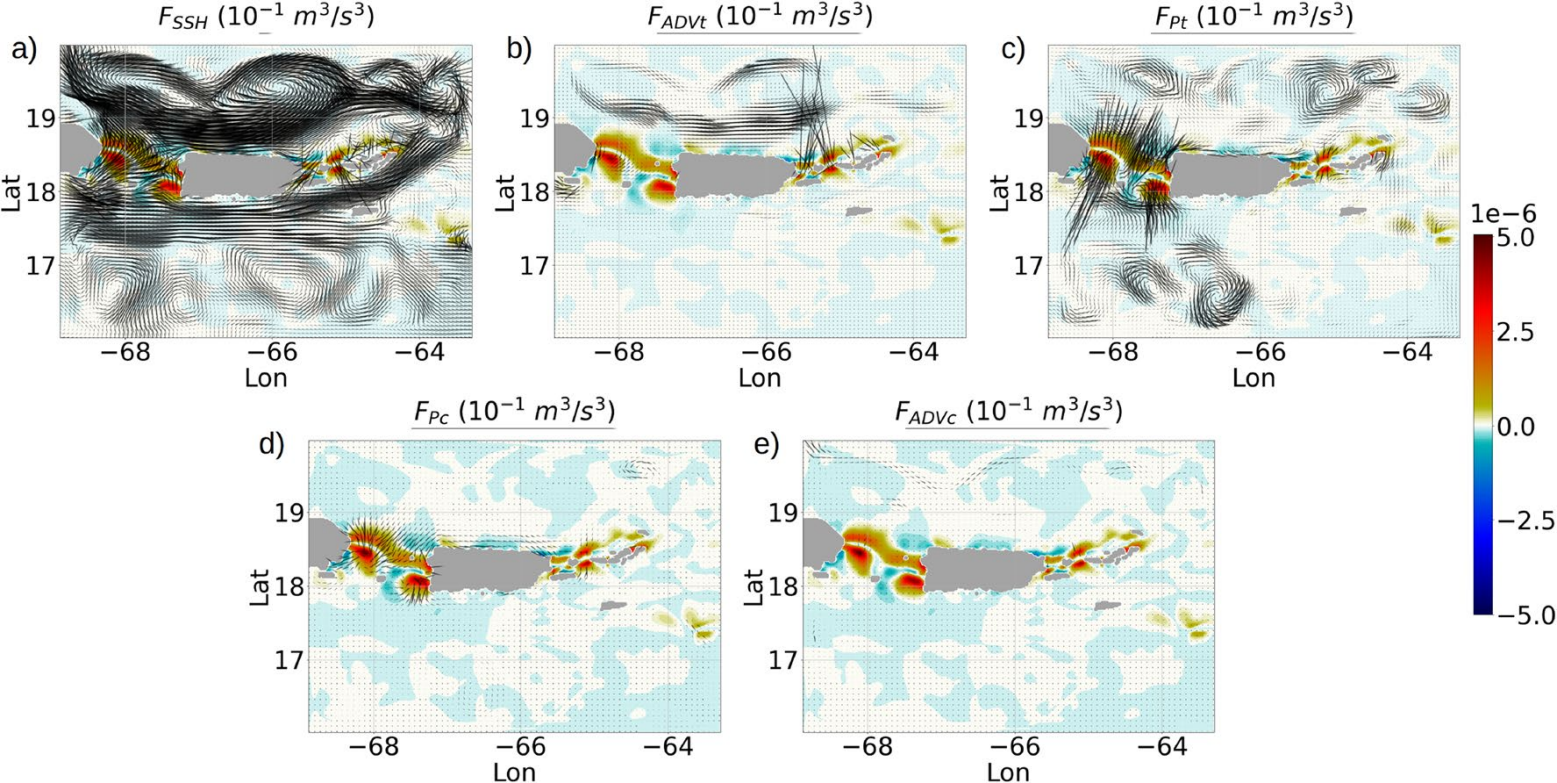
Quiver plots showing the depth-averaged energy fluxes for $E_{BTK}$ (refer to Eq. B11): (**a**) $F_{SSH}$, (**b**) $F_{ADVt}$, (**c**) and $F_{Pt}$, and the depth-averaged energy fluxes for $E_{BCK}$: (**d**) $F_{Pc}$, (**e**) $F_{ADVc}$. The color contours show the depth-averaged conversion term *C*. The arrow lengths denote the flux magnitudes. We preferred to keep the arrow scales different for each plot in order to show the fields prominently. All data are averaged over 1 month to minimize the meso and submesoscale perturbations.

The pressure flux of $E_{BTK}$ due to sea-surface height perturbations ($F_{SSH}$) is typically of O($1~m^3/s^3$) magnitudes (Fig. 8a), and appears to align with the mesoscale and submesoscale meanders (refer to Online Appendix 8 for the energy flux expressions). The advective energy flux $F_{ADVt}$ aligns with the mesoscale meanders in the northern open ocean with typically O($10^{-2}~m^3/s^3$) magnitudes, but shows strongly northward beams of O($1~m^3/s^3$) magnitudes radiating from the passages between St Thomas, Culebra and Puerto Rico (Fig. 8b). The baroclinic pressure energy flux $F_{Pt}$ (Fig. 8c) forms 2 strips of strongly radiating beams from the Mona passage, one directed northward and the other directed southward. In both these strips, the conversion of barotropic to baroclinic tidal energy attains the highest magnitudes of O($10^{-5}~m^2/s^3$). The passage between St Thomas and Culebra also shows northward and southward beams, albeit not as strong as the beams in the Mona passage. The regions where the energy fluxes form strongly northward and southward radiating beams, are characterized by normalized topographic gradient values ranging from 0.4 to 1.0 (Fig. 5a).

#### Baroclinic kinetic energy

In a hydrostatic setup, the components of the depth-averaged $E_{BCK}$ transport are the advective work *ADVc*, baroclinic stress work *Sc*, baroclinic pressure work *Pc*, buoyancy work *Bc*, diffusion term *Dc* and the dissipation $\epsilon _c$ (Eq. B11).

**Figure 9:**
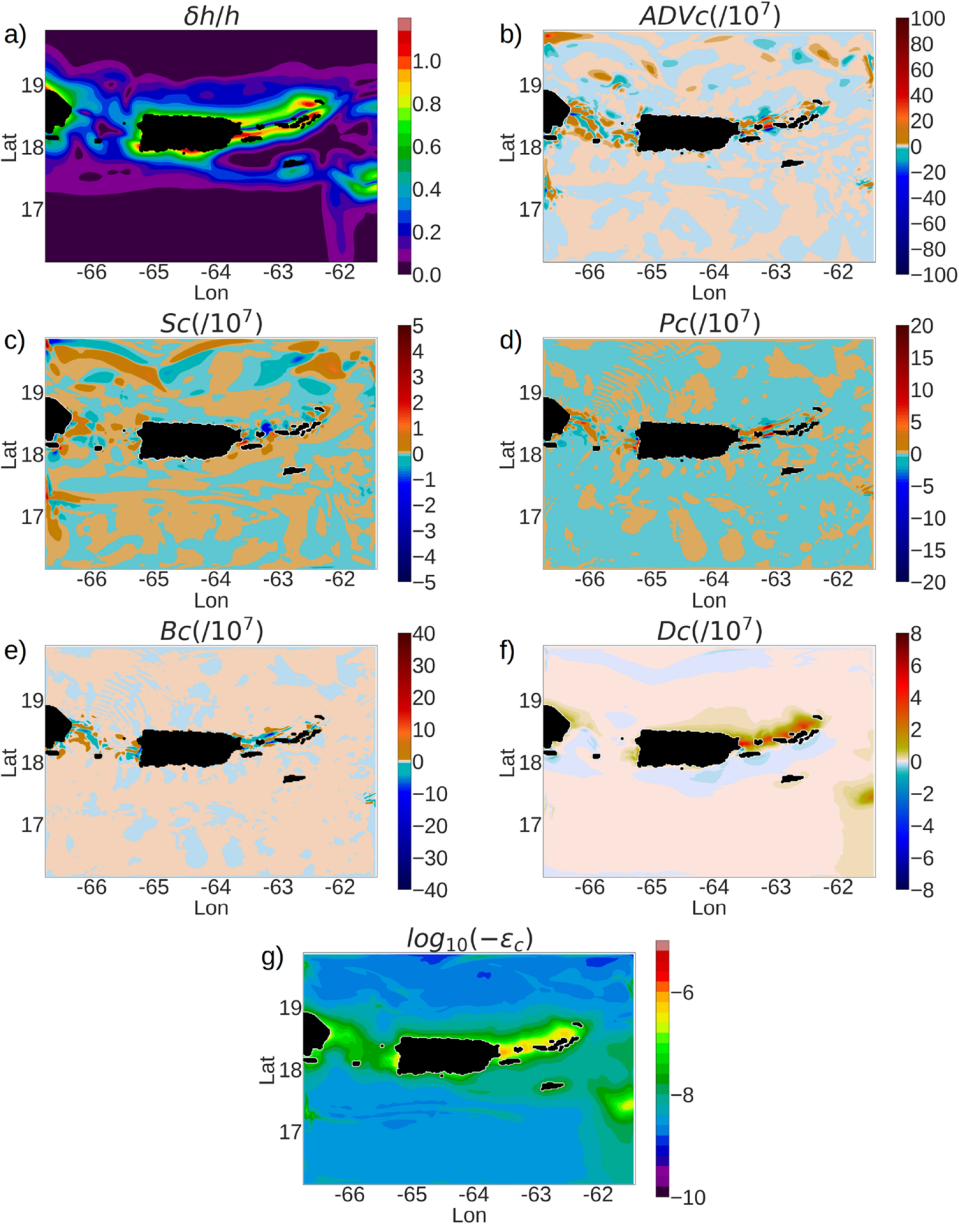
Components of the depth-averaged baroclinic kinetic energy ($E_{BCK}$) Eq. (B11) (see Online Appendix 7) normalized by $10^{-7}$ and averaged over the month of April. (**a**) $\delta h/h$, (**b**) ADVc, (**c**) Sc, (**d**) Pc, (**e**) Bc, (**f**) Dc, and (**g**) $\epsilon _c$. Color palettes show different ranges in each plot in order to display the wave fields prominently. All components are computed in SI units ($m^2/s^3$).

The passages between the islands where $\delta h/h$ ranges from 0.4 to 1.0, show stronger magnitudes of the production, advection, diffusion and dissipation of $E_{BCK}$ compared to the open-ocean regions where $\delta h/h$ is close to 0 (Fig. 9a). The advection term is the strongest in the passages between the islands Culebra, St Thomas and Puerto Rico at magnitudes close to $10^{-5}~m^2/s^3$ (Fig. 9b). Strong magnitudes of advective work also arise in the Mona passage between Puerto Rico and Hispaniola at O($10^{-6}~m^2/s^3$). The pressure work (*Pc*) and buoyancy work (*Bc*) show strong magnitudes of O($10^{-6}~m^2/s^3$) along the northern coastline of the Virgin Islands, and in the passages between Puerto Rico, Culebra and St Thomas (Fig. 9d,e) and are similar in order of magnitude with the baroclinic stress work (Fig. 9c). The diffusion of $E_{BCK}$ holds strongly positive values at O($10^{-7}~m^2/s^3$) along the northern coastline of VI, and weakly negative values along the southern coastlines of VI, Puerto Rico and the southeast of Hispaniola (Fig. 9f). The dissipation of $E_{BCK}$ is close to $10^{-6}~m^2/s^3$ in the passages between Puerto Rico, Culebra and the Virgin Islands, and close to $10^{-9}~m^2/s^3$ in the open-ocean regions (Fig. 9g). In the Mona passage between Hispaniola and Puerto Rico, the depth-averaged dissipation is typically at O($10^{-7}~m^2/s^3$).

The depth-averaged diffusion and dissipation of both $E_{BTK}$ and $E_{BCK}$ in the coastal ocean to the north of the Virgin Islands archipelago is nearly an order of magnitude stronger than that in the southern coastal ocean (Fig. 7g, 9g), and is caused by the contrast between the topographic characteristics of the northern and southern coastal oceans of the archipelago. The northern coastal ocean of the Virgin Islands (figure S2, supplementary material) has a relatively flat topography with a typical depth of −50 m over a spatial extent of 20 km to the north from the island coastlines, whereas the southern coastal ocean topography falls sharply from a depth of −200 m to −1000 m over a spatial extent of 20 km from the southern coastline. Since the surface boundary layer typically varies from −70 m to −20 m from summer to winter, a substantial portion of the vertical water profile in the northern coastal ocean lies within the *ML* where the dissipation is enhanced by surface forcing and submesoscale turbulence. On the other hand, the depth-averaged diffusion and dissipation in the southern coastal ocean of St Thomas and BVI is diminished by the influence of the interior diffusion and dissipation below the *ML*.

**Figure 10:**
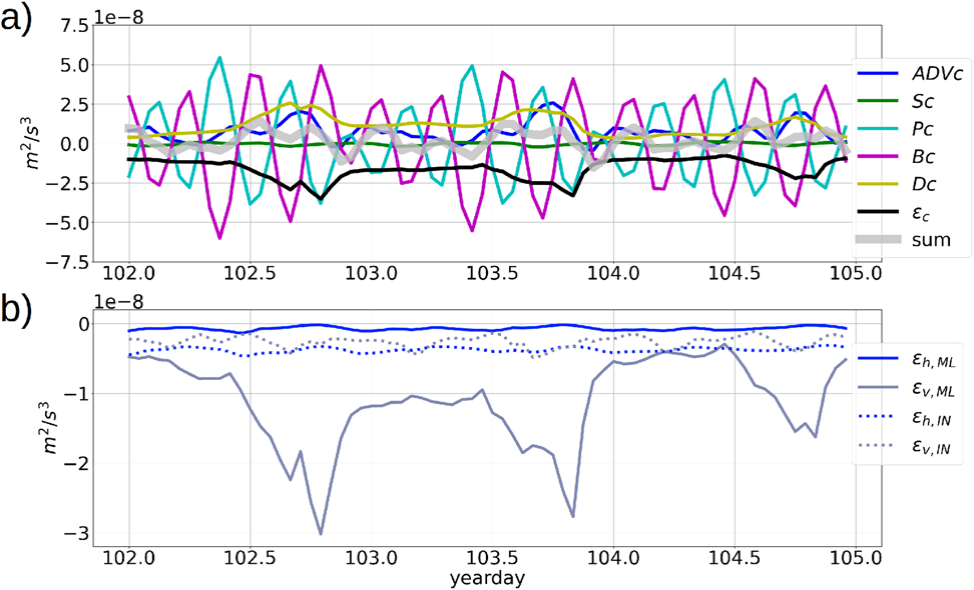
(**a**) Components of the transport equation of the depth-averaged $E_{BCK}$ (Eq. B11) over 3 consecutive days in April, averaged over the entire domain excluding the sponge layer. The gray curve denotes the net tendency, which is the $sum=Pc+Bc+Dc+\epsilon _c - ADVc-Sc$. (**b**) Horizontal ($\epsilon _h$) and vertical ($\epsilon _v$) components of the parameterized dissipation ($\epsilon _c$) of $E_{BCK}$, averaged over the *MLD*, and averaged over the interior depth (*IN*) which is from the topographic depth to the *ML* base.

The baroclinic energy fluxes due to the baroclinic pressure ($F_{Pc}$) show northward and southward beams originating from the Mona passage (Fig. 8d), similar to the divergent beams observed in $F_{Pt}$, but an order of magnitude smaller. The advective flux $F_{ADVc}$ (Fig. 8e) show similar diverging beams that we observed for $F_{ADVt}$ in the region between St Thomas and Culebra, albeit an order of magnitude smaller than $F_{ADVt}$.

A 3-day time-series of the components of the $E_{BCK}$ transport equation (Eq. B11), averaged over the entire domain excluding the sponge layer, shows that the buoyancy production *Bc* negatively correlates with the pressure work (*Pc*) with a coefficient of −0.9 (Fig. 10a). Such strong negative correlation occurs because the vertical component of P ($\overline{-\frac{1}{\rho _0}u_3\frac{\partial p'}{\partial x_3}}$, Eq. B11) is similar in magnitude and opposite in sign to the buoyancy production term $\mathrm {B}=-\frac{g}{\rho _0}\overline{(\rho ' u_3)}$ in a hydrostatic setup. The domain-averaged baroclinic stress work is at O($10^{-9}~m^2/s^3$) and the domain-averaged advection of $E_{BCK}$ is O($10^{-8}~m^2/s^3$). The dissipation ($\epsilon _c$) of $E_{BCK}$ is stronger in magnitude than the diffusion (D) of $E_{BCK}$ and negatively correlates with D with a coefficient of −0.86. The diffusion and dissipation terms are both at magnitudes of O($10^{-8}~m^2/s^3$), and attain a maxima during each day (figure 10a). These maxima are caused by the vertical component $\epsilon _v$ of the dissipation term within the mixed-layer during atmospheric cooling, when the vertical eddy viscosity ($\nu _{ij},~j=3$) is enhanced by the KPP parameterization to account for the mixing due to unstable stratification caused by the cooling (refer to figure S3a,b,c in supplementary material). The domain averaged $\epsilon _c$ includes the contributions of the horizontal and vertical components of $\epsilon _c$ within the *ML* and below the *ML*. A time-variability plot of the individual components of $\epsilon _c$ (Fig. 10b), shows that the strongest contribution to $\epsilon _c$ comes from the vertical component $\epsilon _v$ depth-averaged from the base of the *ML* to the surface. In the interior ocean, both the horizontal and vertical components of $\epsilon _c$ are at O($10^{-9}~m^2/s^3$) in magnitude. The horizontal component of $\epsilon _c$ is larger in magnitude in the interior ocean compared to within the *ML* (figure S4, supplementary material).

### Seasonal variability of tidal energy

**Figure 11:**
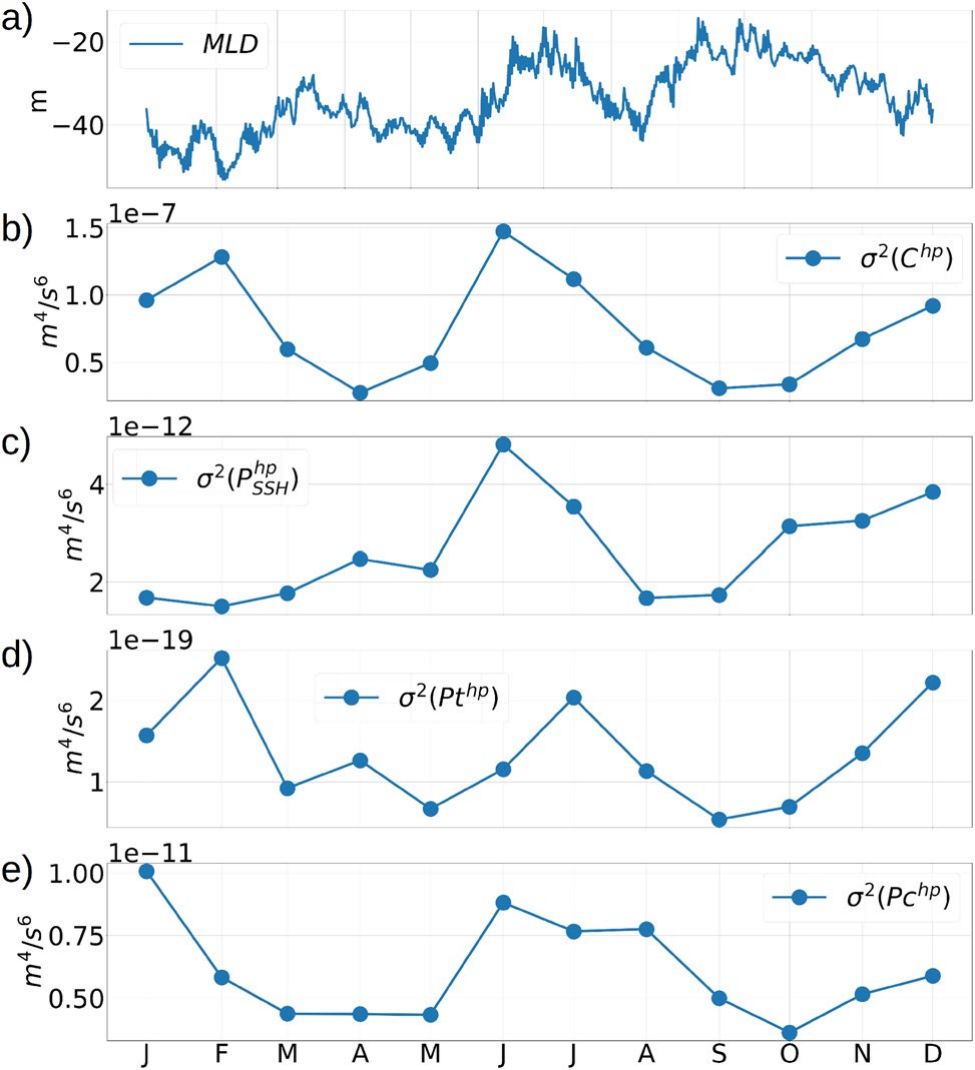
(**a**) The *MLD* averaged over the zonal transect A to the west of St Thomas (transect A shown in figure S5, supplementary material). Plots (**b**), (**c**), (**d**) and (**e**) show the spatial variances ($\sigma ^2$) calculated over transect A for the high-pass filtered depth-averaged conversion term $C^{hp}$, and the pressure transport terms $P_{SSH}^{hp}$, $P_t^{hp}$ and $P_c^{hp}$ respectively. The variances are calculated over transect A at every instantaneous time, and then averaged over each of the 12 months. The monthly averaging was done to show the seasonal variability prominently.

The seasonal changes in surface cooling and warming results in the deepening and shallowing of the *ML* (Fig. 11a). Deeper *ML* during wintertime cooling results in stronger mixing and dissipation which erodes the magnitude of the gradients in the ocean properties, thus reducing the spatial variance. At the same time, deeper *ML* during winter results in higher rates of conversion of submesoscale available potential energy to eddy kinetic energy, resulting in stronger buoyancy gradients due to frontogenesis, and stronger submesoscale fluctuations in the velocities^22,42^. In order to examine the impact of the seasonal variations of the *MLD* on the depth-averaged tidal energy conversion and transport, it is necessary to first extract the tidal signals from the energy components using a high-pass filter.

We calculate the time-series of the depth-averaged conversion rate (*C*) of barotropic to baroclinic energy (Online Appendix 9) and the pressure transport components of $E_{BTK}$ and $E_{BCK}$ (Online Appendix 7) at each individual grid points, and apply a high-pass filter with a cutoff frequency of $1/30~hr^{-1}$ to separate the frequencies corresponding to the diurnal, semi-diurnal and higher-harmonic tidal constituents. The high-pass filtered components of the conversion term *C* and the pressure transport terms $P_{SSH}$, *Pt* and *Pc* are denoted as $C^{hp}$, $P_{SSH}^{hp}$, $Pt^{hp}$ and $Pc^{hp}$ respectively. Plots of the variance of the high-pass filtered conversion rate and pressure transport terms, averaged over each of the 12 months, show a fall in the variance during the winter months from February to May, followed by a rise in variance during summertime stratification from May to July, and a subsequent fall in variance during August to October (Fig. 11b–e). The rise and fall in the variance of the energy transport and conversion terms occur due to a reduction and enhancement in the intensity of the upper-ocean mixing due to the seasonal changes in stratification. Along an open-ocean transect C in the Caribbean Sea (Figure S5, supplementary material), where the depth is −4100 m, the depth-averaged high-pass filtered conversion terms and pressure energy terms show a similar seasonal pattern in their variance, but the ratio of the magnitudes between the 2 transects is typically O($10^3$) for the conversion term and O($10^1$) for the pressure energy terms (figure S6a,b,c,d,e, supplementary material). Note that the topography at transect A is super-critical with normalized gradient $\delta h/h$ values larger than 0.6, whereas the topography at C is sub-critical with $\delta h/h$ close to 0. This implies that the super-critical topography and higher topographic gradients of the continental shelf strengthen the seasonal response in the depth-averaged tidal energy conversion and transport.

**Figure 12:**
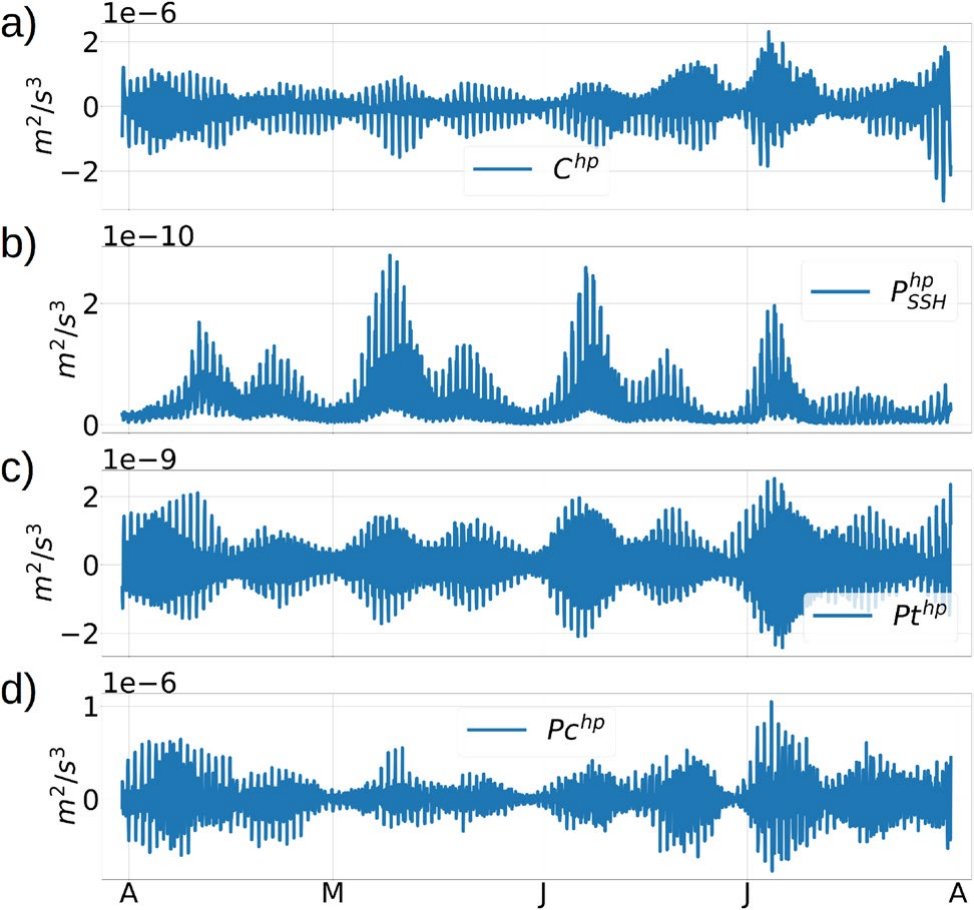
Time-series from April to July, showing the depth-averaged high-pass filtered $C^{hp}$ (plot a), $P_{SSH}^{hp}$ (plot b), $Pt^{hp}$ (plot c), and $Pc^{hp}$ (plot d). All quantities are spatially averaged over the zonal transect A to the west of St Thomas (transect A shown in figure S5, supplementary material).

In the continental shelf to the east of Anegada island, a time-evolution of the high-pass filtered depth-averaged conversion term and the pressure energy transport terms (figure 12a,b,c,d) show a distinct spring-neap variability characterized by 2 maxima each month in the amplitude of the tidal oscillations. The oscillation amplitude is a maximum during the spring tides and a minimum during the neap tides. During the months of July and August, the spring-tidal amplitudes for $C^{hp}$ and $P_c^{hp}$ are noticeably stronger than the amplitudes during the earlier months, which is in agreement with the increase in the variance during the summer months due to the shallowing of the mixed-layer by stronger summertime heating.

### Benthic turbulence near the continental shelf

Turbulence near the topographic depth is enhanced when the bottom friction creates a strong lateral and vertical shear with the underwater current^1,2,47^. The lateral shear can be either cyclonic or anti-cyclonic, which is influential in altering the magnitude and sign of the potential vorticity *q* (Online Appendix 10). The value of *q* taking the opposite sign of *f* is a precursor to various kinds of instabilities which act as routes to the dissipation of turbulent eddy kinetic energy. In the upper ocean, negative stratification during nighttime cooling reverses the sign of *q*, causing gravitational instability and convective mixing^48^. Downfront wind flowing parallel to an upper ocean mixed-layer front forces heavier water over lighter water, reducing *q* to negative values and causing inertial-symmetric and gravitational instabilities^48–50^. While inertial instabilities mostly occur in the upper ocean mixed-layer, observations and numerical simulations of the flow around complex topographic features have shown the formation of deep-water inertial-symmetric and centrifugal instabilities along the sloping topography by anti-cyclonic vortices formed due to topographic drag^1,3,47,51^.

**Figure 13:**
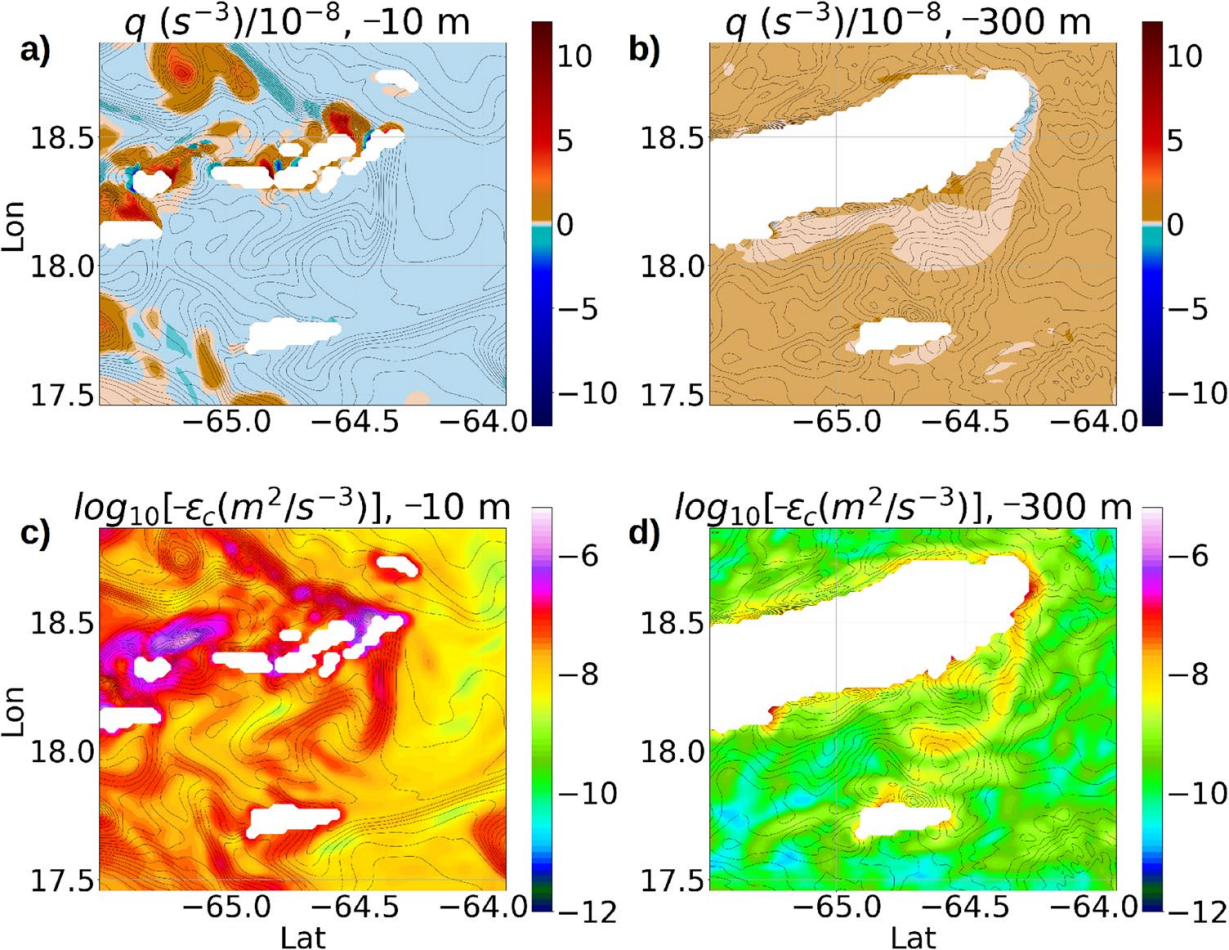
(**a**) Potential vorticity *q* at −10 m. (**b**) *q* at −300 m. (**c**) $\epsilon _c$ at −10 m. (**d**) $\epsilon _c$ at −300 m. All plots are prepared at yearday 273, 00:00 am. The white regions lie within the landmass.

Plan view of the simulated potential vorticity *q* at a depth of −10 m (Fig. 13a) shows mostly weak negative *q* close to 0 throughout the domain due to unstable stratification, and localized regions with intensified positive *q* along the northern coastline of the islands where the dissipation of $E_{BCK}$ is almost 3 orders of magnitude higher than the open ocean dissipation at the same depth (Fig. 13c). Even though negative *q* leads to enhanced dissipation initiated by inertial instabilities, enhanced dissipation also happens in the presence of positive *q* initiated by barotropic lateral shear instability^51^ and ageostrophic baroclinic instability due to the slumping of isopycnal surfaces^36,37^.

At a depth of −300 m, the values of *q* are positive and close to 0 almost throughout the entire region. However, negative *q* appears along the eastern edge of Anegada and St Croix (Fig. 13b) and get advected southward, forming a continuous streak of turbulent water with dissipation levels at almost 3 orders of magnitude higher than the open ocean dissipation at the same depth (Fig. 13d). These turbulent streaks characterized by negative *q* originating along the edges of the islands are O(10 km) long, and occur up to a depth of −400 m in the continental shelf (figure S7a,b in supplementary material).

**Figure 14:**
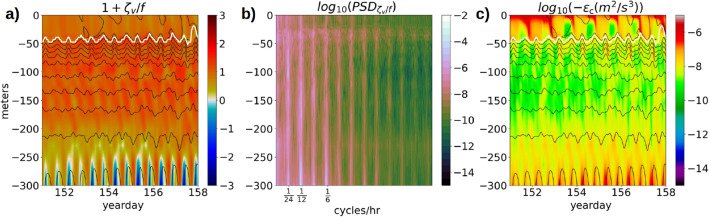
(**a**) Vertical profile of $1+\zeta _v/f$ in the continental shelf near the eastern edge of Anegada over 7 days starting at May 1st 00:00 hr. (**b**) PSD of $\zeta _v/f$ for a 1-month time series in May at the same location. (**c**) Dissipation $\epsilon _c$ at the same location for 7 days

In a positively stratified water column, the reversal of sign of *q* happens in an anticyclonic flow where the absolute vorticity $f+\zeta _v$ is strongly negative such that the vertical component of *q* [$(f+\zeta _v)N^2$] (Eq. F1) exceeds the horizontal component ($-\frac{\partial v}{\partial z}\frac{\partial B}{\partial x} + \frac{\partial u}{\partial z}\frac{\partial B}{\partial y}$) in magnitude. Along a vertical profile close to the eastern edge of Anegada, where the topography is −320 m deep (Fig. 14a), the time-series of the normalized absolute vertical vorticity ($1 + \zeta _v/f$) shows a periodic formation of anticyclonic vortices with O(1) magnitudes near the benthic depth. The periodicity of this formation is attributed to the oscillatory characteristics of the tidal currents with diurnal, semi-diurnal and higher-harmonic frequencies that are noted in the PSD plots of $\zeta _v/f$ at the same location (Fig. 14b). Due to the formation of these anti-cyclonic vortices with strongly negative magnitudes, the *q* reverses sign, resulting in inertial-symmetric instabilities that lead to submesoscale turbulence, enhancing the dissipation magnitudes to O($10^{-6}~m^2/s^3$) near the benthic depths (Fig. 14c).

**Figure 15:**
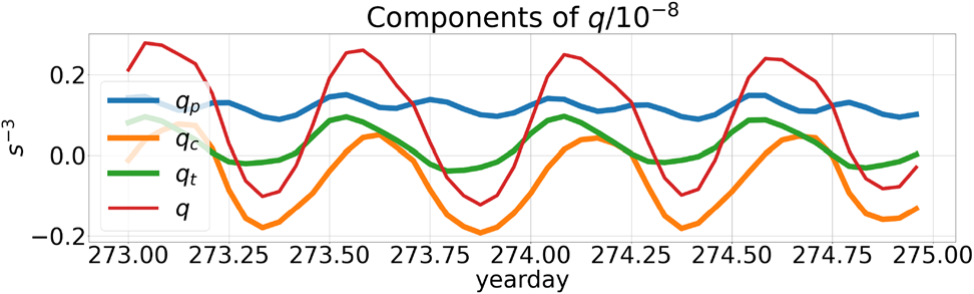
Time-series of the components of *q* at the first $\sigma$ surface in the continental shelf near the eastern edge of Anegada (depth −300 m).

**Figure 16:**
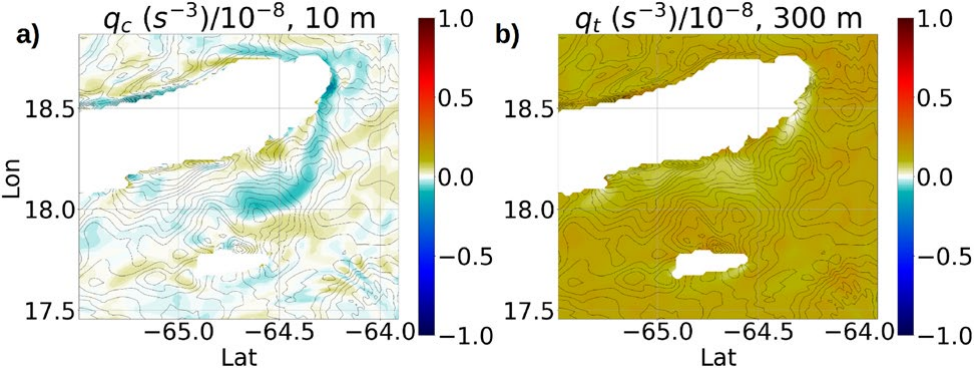
Snapshots at −300 m depth showing (**a**) instantaneous baroclinic component ($q_c$) of the potential vorticity at yearday 273, 00:00 AM; (**b**) barotropic component ($q_t$) at the same time. Expressions for the baroclinic and barotropic components of *q* are shown in Online Appendix 10.

The potential vorticity *q* is a summation of the inertial component $q_p$ caused by planetary rotation, the baroclinic component $q_c$ and the barotropic component $q_t$ (Eq. F3 in Online Appendix 10). Near the eastern edge of Anegada island, a time-evolution plot of the components of *q* on the 1st $\sigma$ surface from the bottom shows that all the three components of *q* exhibit periodic oscillations caused by tides (Fig. 15). The components $q_c$ and $q_t$ both acquire periodically negative values with $q_c$ forming stronger fluctuations than $q_t$. A plan view of the components of *q* at a depth of −300 m (Fig. 16a,b) reveals that while the negative $q_t$ is confined only to a localized region along the eastern edge of Anegada, the negative $q_c$ forms O(10 km) long streaks covering a much larger surface area throughout the domain at the given depth.

**Figure 17:**
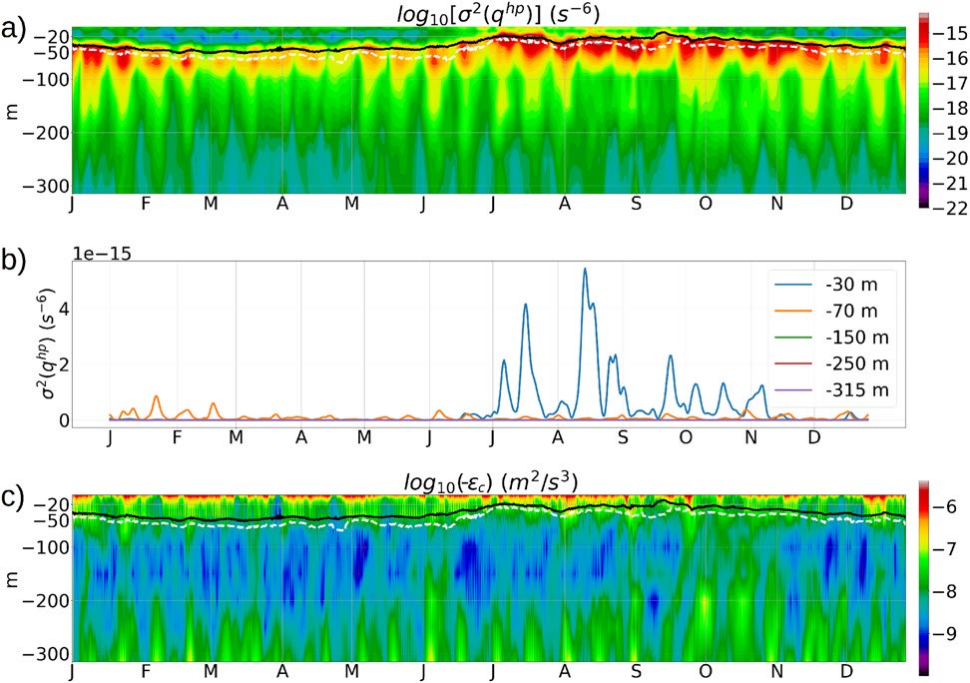
(**a**) Variance ($\sigma ^2$) of the high-pass filtered potential vorticity ($q^{hp}$) at a location in transect B to the east of Anegada (transect B shown in figure S5, supplementary material). The black line shows the *MLD*, and the dashed white line denotes the depth of maximum vertical stratification in the water column (transition-layer depth^52^). (**b**) Time-series of $\sigma ^2(q^{hp})$ at different depths. (**c**) Dissipation $\epsilon _c$ at the same location in the transect B.

In order to study the impact of seasonal changes in the upper-ocean stratification on the tidal oscillations in *q*, we first extract the tidal oscillation component of *q* by applying a high-pass filter to the time-series of *q* at each grid point with a cutoff frequency of $1/30~hr^{-1}$. A contour plot of the variance ($\sigma ^2$) of the high-pass filtered potential vorticity ($q^{hp}$) along a meridional transect at the eastern edge of Anegada (transect B in figure S5, supplementary material), shows that at any instantaneous time, the strongest tidal oscillations in the vertical water column of *q* occur at the depth of the maximum stratification in the water column, also called the transition-layer depth^52^ (Fig. 17a). During the winter months from January to May, the magnitudes of $\sigma ^2(q^{hp})$ within the *ML* are the lowest in the range of $10^{-20}~s^{-6}$ to $10^{-18}~s^{-6}$, and at $10^{-16}~s^{-6}$ at the transition-layer depth. As the *ML* shallows to −20 *m* from June to September, the $\sigma ^2(q^{hp})$ at the transition-layer depth increases to O($10^{-15}~s^{-6}$), followed by deepening of the *ML* to −50 m during the last 2 months of the year. Plots of $\sigma ^2(q^{hp})$ at specific depths show a prominent seasonal variability at a depth of −30 m but no seasonal variability at the depths −70 m and deeper (Fig. 17b). A contour plot of the averaged dissipation of $E_{BCK}$ at the same transect (Fig. 17c) shows that the strongest dissipation along the water column is confined within the top −20 *m* from the surface, which lies within the *ML*. There is also enhanced dissipation of O($10^{-7}~m^2/s^3$) over a depth range of 50 *m* from the bottom, which is caused by the lateral shear produced by the tidal oscillations.

**Figure 18:**
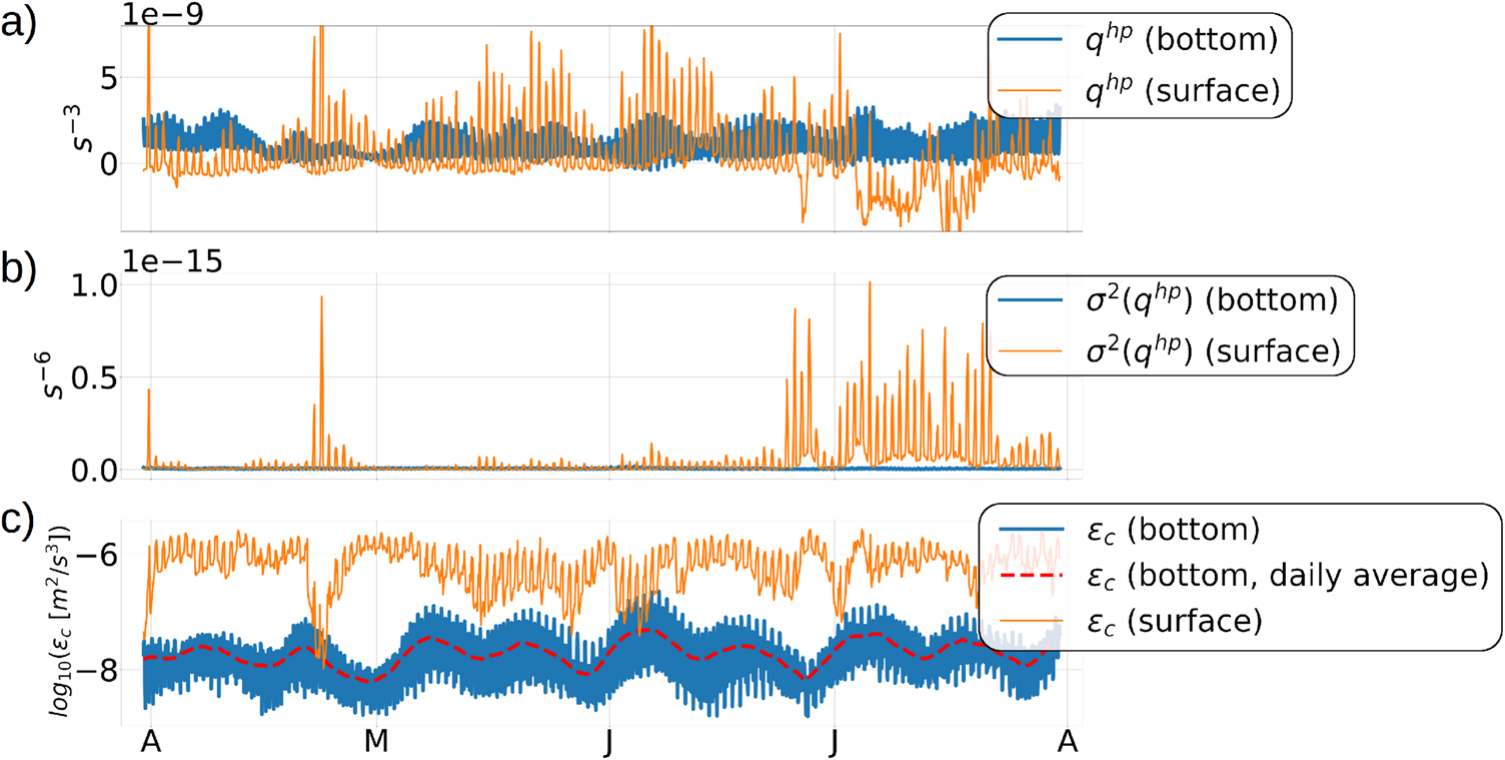
Hourly time series of the (**a**) high-pass filtered potential vorticity $q^{hp}$ near the surface and bottom, (**b**) variance of $q^{hp}$ over transect B near the surface and bottom, and (**c**) dissipation $\epsilon _c$ near the surface and bottom near the eastern edge of the Anegada island (where the depth is −320 m). The dashed red line is the daily averaged benthic dissipation.

Hourly time-series of $q^{hp}$ and the baroclinic kinetic energy dissipation $\epsilon _c$ in the continental shelf near the eastern edge of Anegada show a distinct spring-neap pattern near the benthic depth of −320 *m* (Fig. 18a, c). We notice a contrast between the responses of the benthic $q^{hp}$ and the benthic $\epsilon _c$ to the spring-neap tidal signals. For the benthic $q^{hp}$, the amplitude of the daily tidal fluctuations is enhanced during the spring tides and weakened during the neap tides. In contrast, it is the daily-averaged benthic $\epsilon _c$ (dashed red line, Fig. 18c) that responds to the spring-neap signals, whereas the daily tidal fluctuations in the benthic $\epsilon _c$ remain unaffected by the spring-neap signals. The near-surface $\epsilon _c$ and $q^{hc}$ both exhibit stronger magnitudes than the benthic $\epsilon _c$ and $q^{hc}$ but do not show a spring-neap pattern in their time-series, which indicates that the near-surface potential vorticity and dissipation are more responsive to the upper-ocean processes like submesoscale turbulence, wind forcing, and heating and cooling. The seasonal change in the stratification from May to July impacts the tidal oscillation amplitudes of the near-surface $q^{hc}$ resulting in an increase in it’s variance by 4 orders of magnitude (Fig. 18b).

In the open-ocean water column, the time-series and PSD of $\zeta _v/f$ show tidally forced oscillations in $\zeta _v/f$ along the vertical profile (figure S8a,b,c and S9a,b,c in supplementary material). The spectral amplitudes of the PSD of $\zeta _v/f$ in the open ocean are not as strong as the ones near Anegada, which is in agreement with our inference from the “Kinetic energy analysis” section that majority of the internal tidal kinetic energy is produced over the steep topographic slopes close to the islands. Since the tidal oscillations in $1+\zeta _v/f$ at the benthic depth are not strong enough to cause inertial instability by reversing the sign of *q*, the simulated dissipation in the open ocean benthic depths does not reach O($10^{-6}~m^2/s^3$) magnitudes (figure S9c, supplementary material).

### Variability of benthic turbulence on tidal parameters

**Figure 19:**
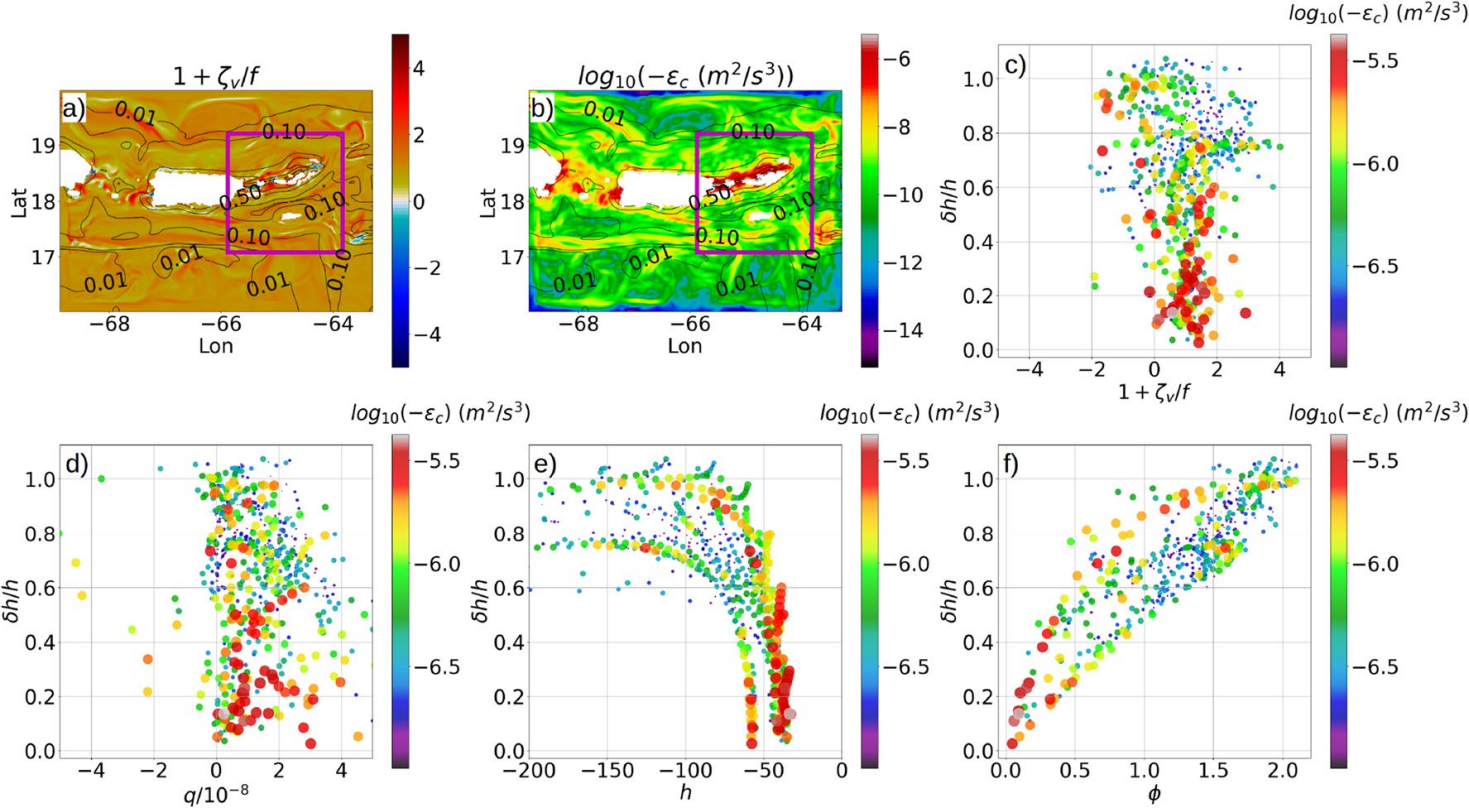
(**a**) Snapshots of normalized absolute vorticity ($1+\zeta _v/f$) and (**b**) the dissipation $\epsilon _c$ on the first $\sigma$ surface, averaged over 1 day at yearday 273, 00:00 hr. The black contours are $\delta h/h$. The purple rectangle is the Virgin islands subdomain. Scatter plots of (**c**) $1+\zeta _v/f$ with $\delta h/h$, (**d**) *q* with $\delta h/h$, (**e**) topographic depth *h* with $\delta h/h$, (**f**) tidal steepness parameter $\phi$ with $\delta h/h$. All scatter plots are computed on the 1st $\sigma$ surface and averaged over 1 day within the Virgin islands subdomain. The color palette shows the dissipation levels. The scatter marker size varies with the magnitude of $log_{10}(-\epsilon _c)$.

At the first $\sigma$ layer of the vertical grid, the regions along the northern coastline of St Thomas, St John and BVI (excluding Anegada) show strongly cyclonic absolute vorticity ($1+\zeta _v/f$) values at O(1), and strong levels of dissipation at O($10^{-6}~m^2/s^3$) (Fig. 19a, b). Along the southern coastline of the Virgin Islands, $1+\zeta _v/f$ is mostly cyclonic and weaker in magnitudes compared to the northern region, and the dissipation levels are nearly 2 orders of magnitude weaker than the northern region. In the Mona passage, the dissipation levels are at O($10^{-6}~m^2/s^3$) along the eastern coastline of Hispaniola and the western coastline of Puerto Rico.

While the absolute vorticity in the coastal ocean around the Virgin Islands is predominantly cyclonic, anti-cyclonic absolute vorticity occurs at a few specific locations in between the islands (Fig. 19a); between Anegada and Virgin Gorda, Culebra and Vieques, and the western edge of St Thomas. These regions of strongly turbulent water with O(1) cyclonic and anti-cyclonic vortical nature, are located near steep topographic slopes with normalized gradient values ranging from 0.5 to 1.0 as indicated by the labeled black contour lines in Fig. 19a.

The sign of the absolute vorticity near the topographic depth depends on the orientation of the flow, whereas the strength of the vorticity depends on the magnitude of the flow. Strong velocity amplitudes associated with tidal oscillations strengthen the lateral shear close to the topography due to bottom drag, thereby intensifying the vertical vorticity. If the topographic slope is to the left of the flow direction, cyclonic vortices are likely to occur and lead to barotropic lateral shear instability. If the topographic slope is to the right of the flow direction, anti-cyclonic vortices are likely to form, leading to inertial instability. In both cases, secondary shear instabilities emerge^53^ and pave the way to submesoscale turbulence and mixing^1^. Although the coarse resolution of our model makes it impossible to resolve the secondary shear instabilities, the mixing parameterizations used by the USCROMS are able to crudely represent the enhanced dissipation associated with the turbulence.

In the deep open ocean, where $\delta h/h$ is typically less than 0.01, the USCROMS outputs at the first $\sigma$ surface show long streaks with dissipation levels as high as O($10^{-7}~m^2/s^3$) and strongly cyclonic O(1) values of $1+\zeta _v/f$ (Fig. 19a, b). However, since the baroclinic tidal current velocities are weaker in magnitude in these regions compared to the passages close to the islands, the intensity of lateral shear, and consequently the dissipation in these regions, is weaker than in the locations close to the islands. Moreover, since the terrain-following $\sigma$ levels render a coarse vertical resolution at the $\sigma$ grid close to the open-ocean topography, a realistic estimate of the dissipation in the benthic boundary layer of the deep open ocean cannot be realized in this simulation. Numerical models with higher vertical and horizontal resolution near the topography are necessary to realistically estimate the bottom dissipation variability in the deep open ocean. Analysis of the dissipation of $E_{BCK}$ near benthic depths in the Caribbean Sea using high resolution USCROMS nested grids is currently underway at UVI.

Scatter plots of the normalized absolute vorticity $1+\zeta _v/f$ and the normalized topographic gradient $\delta h/h$ in the Virgin Islands subdomain (Fig. 19c) show that strong dissipation levels, indicated by red dots, occur in the regions with both positive and negative values of $1+\zeta _v/f$. The dots with negative $1+\zeta _v/f$ are less in number than those with positive $1+\zeta _v/f$, and mostly coincide with the higher values of $\delta h/h$ from 0.6 to 1.0 and strong dissipation levels at O($10^{-6}~m^2/s^3$). The regions with positive $1+\zeta _v/f$ are found throughout the entire range of $\delta h/h$ from 0 to 1.0, however, the strongest dissipation levels indicated by the red dots occur at the lower values of $\delta h/h$ from 0 to 0.5. These regions with the strongest dissipation levels also show positive *q* (Fig. 19d) and depths ranging from −60 m to −30 m (Fig. 19e), and with steepness parameter values within 0 to 0.5 (Fig. 19f), implying that the mixing and dissipation in these regions is enhanced by the surface-forced processes like wind forcing, mixed-layer instabilities, bottom friction and nighttime convective cooling^48^. Within a depth-range of −150 m to −70 m, we observe a number of yellow and orange dots with steepness parameter values greater than 1.0 (Fig. 19f), $\delta h/h$ greater than 0.8, and strong dissipation at levels greater than O($10^{-6}~m^2/s^3$). Most of these dots have positive values of *q* (Fig. 19d), and a few dots show negative *q*. The ones with positive *q* indicate barotropic shear instability, and the ones having negative *q* indicate inertial instability.

From the above analysis, we infer that if the topography is shallow enough to lie within the surface mixed-layer, the benthic dissipation levels are enhanced by the surface-forced processes. If the topography lies below the mixed-layer, the factors governing the benthic dissipation levels are the normalized topographic gradient, the tidal steepness parameter, and the magnitude and orientation of the flow. At the regions within the continental shelf where the topography is super-critical with normalized topographic gradients close to 1.0, a strong conversion of barotropic to baroclinic tidal energy leads to strong oscillations in the baroclinic current velocities. These oscillations periodically enhance the lateral shear leading to the formation of inertial and lateral shear instabilities depending on the orientation of the flow. The lateral and inertial instabilities extract energy from the baroclinic tidal kinetic energy and feed it into turbulence, leading to strong dissipation at 3 orders of magnitude higher levels than the open ocean benthic dissipation.

## Conclusion

Using numerical modeling, we study the physical characteristics of the US Caribbean ocean in context to the seasonality in the upper-ocean submesoscale turbulence, and the internal tides generated by the complex coastal topography. We use the USCROMS modeling system for our study, which is the ROMS AGRIF model configured with the US Caribbean regional climatology and local atmospheric forcing.

The USCROMS outputs show that the submesoscale vertical velocity magnitude is typically O(10 m/day) in the mixed-layer, with the strongest magnitudes near 50 *m*/*day*. The simulated US Caribbean ocean exhibits a seasonal submesoscale cycle characterized by shallowing of the mixed-layer from a typical depth of −70 m during winter to −20 m in the summer. The vertical velocity fluctuations are stronger during winter and weaker during summer, as is the case with submesoscale seasonality. The normalized relative vorticity magnitudes during winter months attain O(1) values due to intensified submesoscale motions, whereas during the summer months it is reduced to near 0. The contrast between the submesoscale strength during winter and summer is attributed to the higher rates of conversion of available frontal potential energy to eddy kinetic energy in a deeper mixed-layer, a phenomenon that had been observed in various upper ocean measurements and demonstrated in numerical studies using submesoscale resolving models.

Internal tides originate from the passages between the islands of St Thomas, Culebra and Puerto Rico, the Mona passage between Hispaniola and Puerto Rico, and the passage between Anegada and Virgin Gorda. These regions, characterized by super-critical topography and depths in the range of −100 m to −400 m, exhibit the strongest conversion rates of the depth-averaged barotropic to baroclinic tidal energy, and are associated with the strongest tidal oscillations compared to the open-ocean regions. The semi-diurnal internal tide is found to be the most energetic internal tide emerging from these regions. Apart from the diurnal and semi-diurnal internal tides, non-linear internal waves at higher-harmonic frequencies emerge from several locations close to the islands where the topography is super-critical.

The strongest conversion rates of the barotropic to baroclinic tidal energy are noted in the continental shelf close to the islands with super-critical topography and depths in the range of −100 m to −400 m: the Mona passage between Hispaniola and Puerto Rico, the passages between St Thomas, Culebra and Puerto Rico, and between Anegada and Virgin Gorda. In these regions, the baroclinic tidal kinetic energy is produced from barotropic tidal kinetic energy through the buoyancy production, and also by the extraction of energy from the barotropic shear by the baroclinic stress divergence. The tidal kinetic energy produced in these regions is advected at an order of magnitude stronger rates than the rates of production. The depth-averaged diffusion and dissipation at the locations with super-critical topography are nearly 3 orders of magnitude stronger than the open ocean dissipation.

At the regions close to the islands with super-critical topography, the depth-averaged conversion rates and tidal kinetic energy transport exhibit a strong response to the seasonal changes in the stratification. Enhanced upper-ocean mixing and dissipation during winter months lowers the magnitude of the depth-averaged conversion rates of the barotropic to baroclinic tidal energy and the transport rates of the baroclinic tidal kinetic energy. Likewise, summertime stratification enhances the conversion and tidal kinetic energy transport rates due to weakened mixing and dissipation. The open ocean conversion rates and energy transport also show a seasonal response, however, the strength of the open-ocean seasonal response is weaker compared to the seasonal response in the continental shelf with super-critical topography.

At the benthic depths, the characteristics of turbulence and dissipation caused by tidal motions varies with the topographic characteristics. At the regions where the topographic depth is shallower than the transition-layer depth, the potential vorticity (*q*) and dissipation magnitudes are strongly responsive to the seasonal changes in the stratification. If the topographic depth lies below the transition layer, the benthic dissipation of tidal kinetic energy and potential vorticity are not affected by the surface-forced processes or the seasonal changes in stratification, but only by the spring-neap tidal variability. The transition from the neap to spring tides occurs twice a month, resulting in an increase in the amplitude of the daily tidal fluctuations in the benthic potential vorticity on every spring tide, while also periodically enhancing the daily averaged benthic dissipation levels with every spring tide.

At the benthic regions with steep super-critical topographic slopes in the continental shelf, the dissipation of the baroclinic kinetic energy is strengthened by periodically occurring inertial instabilities and lateral shear instabilities. Inertial instability is initiated by the reversal of sign of the potential vorticity, whereas lateral shear instability occurs in the presence of positive potential vorticity and weak stratification^1^. The oscillatory motion of the barotropic and baroclinic tidal currents in the vicinity of the sloping topography facilitates the periodic formation of strongly anti-cyclonic vortices with negative *q* values and O(1) Rossby numbers. The contribution of the baroclinic tidal oscillations, by magnitude, is more influential to the formation of negative *q* than the barotropic oscillations. The anti-cyclonic vortices with negative *q* get advected by the periodically oscillating currents, forming O(10 km) streaks of turbulent water with nearly 3 orders of magnitude stronger dissipation levels than the open-ocean dissipation at the same depth. The passages between Anegada and Virgin Gorda, between St Thomas, Culebra and Puerto Rico, and localized regions along the coastline of Hispaniola and the western coastline of Puerto Rico form inertial instabilities at the benthic depths by the reversal of sign of *q*, enhancing the local dissipation levels to 3 orders of magnitude stronger than the open-ocean dissipation at the same depth.

Enhanced dissipation also occurs near the benthic depths in the open ocean at the regions where the absolute vorticity is strengthened by the shear caused by topographic friction. However, since the magnitude of the baroclinic tidal currents in the deeper ocean is not as strong as the currents near the islands, the lateral shear and consequently the dissipation in the benthic open ocean is not strong enough to create the high levels of dissipation occurring near the islands. The contrast between the tidal oscillation amplitudes in the island passages and in the deep open ocean is because majority of the baroclinic tidal kinetic energy originates in the island passages and is dissipated quickly while propagating toward the open ocean.

This study has a few caveats. Simulating the spatial variability of benthic dissipation by internal tides requires adequate vertical resolution to estimate the turbulence in the bottom boundary layer caused by the oscillating tidal currents. The $\sigma$ layers of the parent grid of the USCROMS system render a vertical resolution near the topography that varies from 10 m near the islands to 250 *m* in the open ocean (figure S1, supplementary material). Therefore, the parent grid of our system is unable to simulate a realistic bottom boundary layer in the open ocean, which is a limitation to this study. Moreover, the 2 km horizontal resolution of the parent grid can only partially resolve inertial-symmetric instabilities^54^, and cannot resolve the subsequent Kelvin-Helmholtz instabilities that equilibriate the negative potential vorticity^53^. Coarse resolution is also unable to represent the complex coastal topographic features close to the islands. Barotropic tidal sloshing over such sharp topographic features results in non-linear internal-wave breaking and dissipation, which can be only resolved by fine-resolution numerical models^55^. Therefore, finer resolution nested grids in non-hydrostatic mode are needed to understand the dynamics of regional internal tide production, and its impact on the inertially unstable water bodies forming along the continental shelf topography. Studying the local physical aspects of the tides, currents and their interaction with the US Caribbean topography using high resolution nested grids is currently underway at UVI.

## Supplementary Information

Supplementary Information 1.
