# Supplementary material for: Numerical modeling of internal tides and submesoscale turbulence in the US Caribbean regional ocean
Source: Sci Rep. 2023 Jan 19;13:1091. doi: 10.1038/s41598-023-27944-2 (PMC9852596; doi:10.1038/s41598-023-27944-2)
Supplement: Supplementary file 1 — Supplementary Information 1. [file 41598_2023_27944_MOESM1_ESM.pdf]

Supplementary material and appendices

Sonaljit Mukherjee

This supplementary material contains the appendices, and some additional plots provided along with the submitted manuscript.

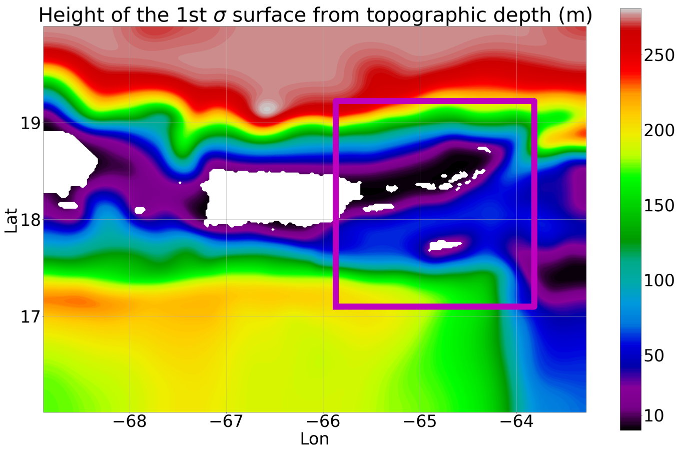

Figure S 1 Height of the first σ surface with respect to the topographic depth. The area bounded by the rectangle is the Virgin Islands subdomain.

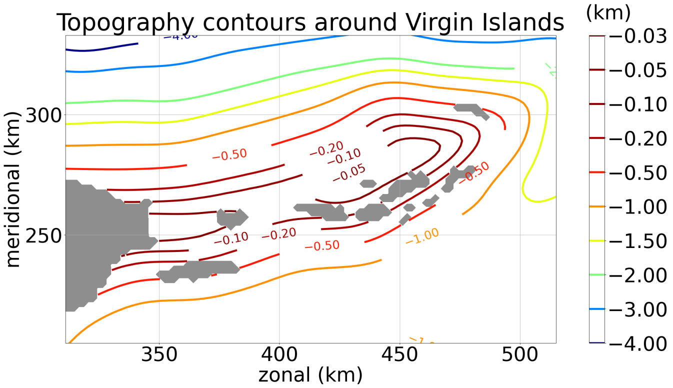

Figure S 2 USCROMS parent grid topography, zoomed over the Virgin Islands region. Contour lines indicate the depth in km.

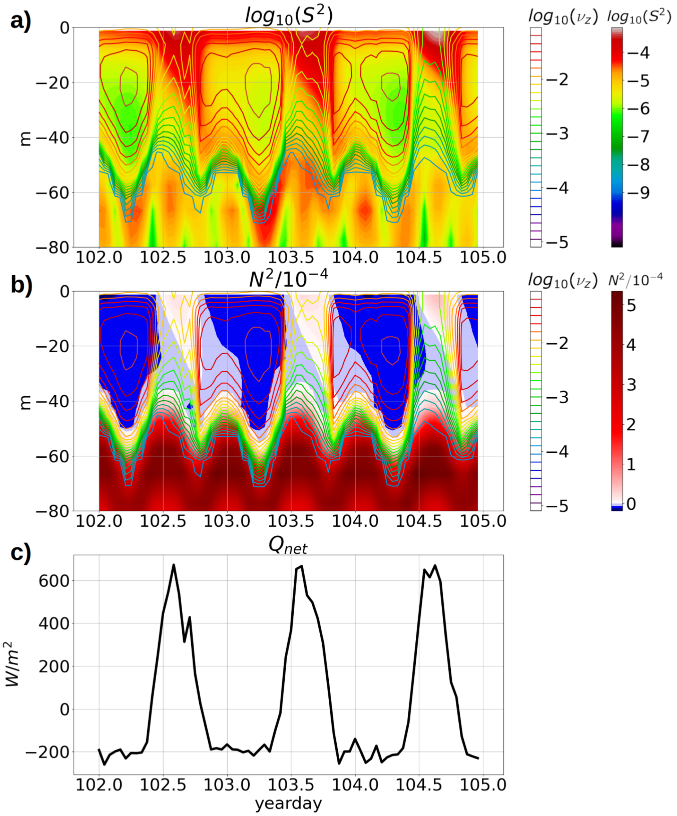

Figure S 3 Time-variability of shear squared (S^2) (plot a), buoyancy frequency N^2 (plot b) and the net surface heat input Q_{net} (plot c) over 3 days in April. The contour lines in plots (a) and (b) denote the vertical eddy viscosity.

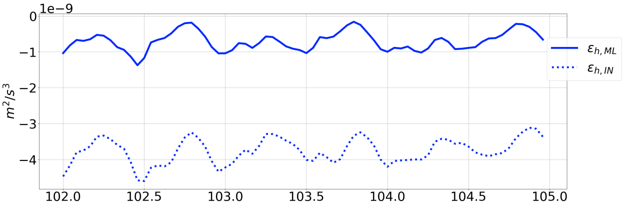

Figure S 4 Time variability of the horizontal component of the dissipation, depth-averaged from the base of the mixed-layer to the surface (solid line), and from the bottom depth to the base of the mixed-layer (dashed line). The x-axis shows the yearday for 3 days in April.

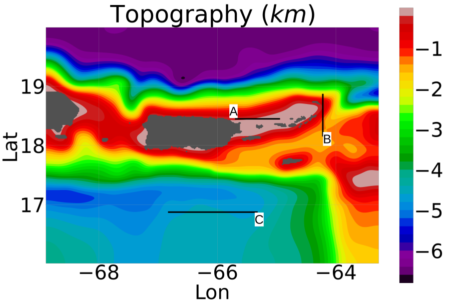

Figure S 5 Topographic depth of the USCROMS domain, with 3 transects marked A, B and C. We conduct analysis of the tidal energy transport terms along these 3 transects.

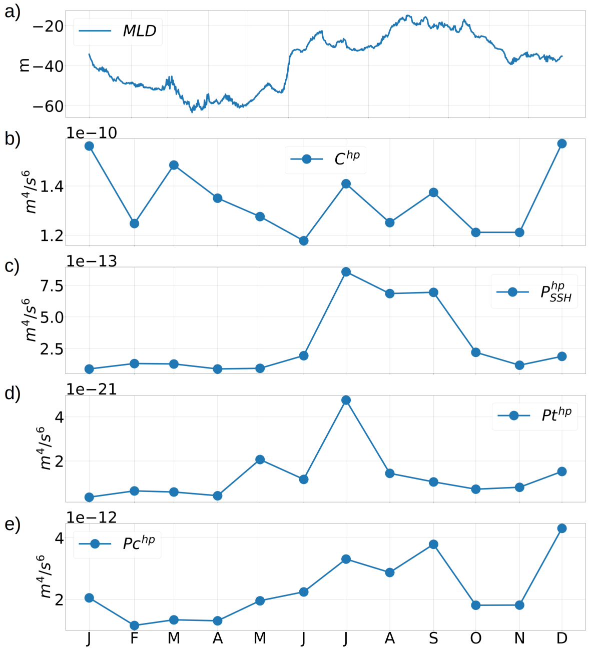

Figure S 6 (a) Averaged MLD over transect C (shown in figure 7, supplementary material) in between BVI and Anegada. Plots (b), (c), (d) and (e) show the spatial variances calculated over transect C (shown in supplementary material figure S5) for the high-pass filtered conversion term C^{hp}, and the pressure transport terms P^{hp}_{SSH}, P^{hp}_{t}, P^{hp}_{c} respectively. These variances are calculated instantaneously, and then averaged over each of the 12 months. The monthly averaging was done to show the seasonal variability prominently.

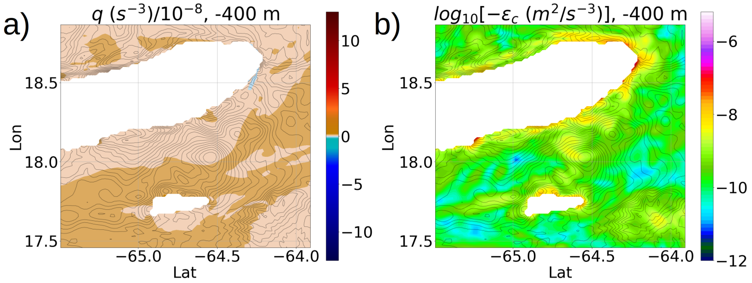

Figure S 7 (a) Potential vorticity q at -400 m. (b) Dissipation ϵ_c at -400 m. All plots are prepared at yearday 273, 00:00 am. The white regions lie within the landmass.

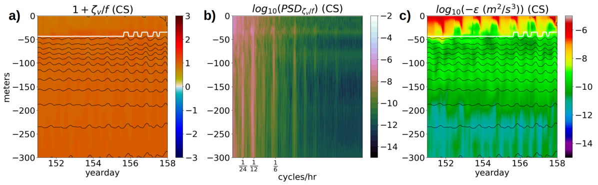

Figure S 8 (a) Vertical profile of $1 + \zeta_v/f$ over 7 days starting at May 1st 00:00 hr, at the location 16.9^0 $N, -64.35^0$ W in the Caribbean sea (where the depth is -3000 m); (b) PSD of ζ_v/f for a 1-month time series in May at the same location; (c) dissipation of baroclinic kinetic energy at the same location for 7 days.

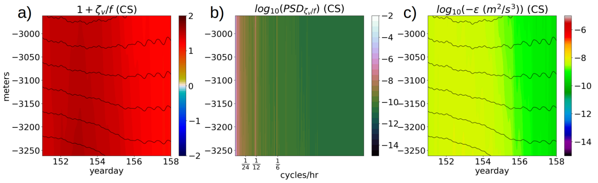

Figure S 9 (a) Vertical profile of $1 + \zeta_v/f$ over 7 days starting at May 1st 00:00 hr, at the same location in figure S8, zoomed over a depth range of 300 m from the bottom topography; (b) PSD of ζ_v/f for a 1-month time series in May at the same location and depth range; (c) dissipation of baroclinic kinetic energy at the same location and depth range for 7 days.

References

[1] Garrett, C., Kunze, E.: Internal tide generation in the deep ocean. Annu. Rev. Fluid Mech. **39**, 57–87 (2007)

[2] Kang, D., Fringer, O.: Energetics of barotropic and baroclinic tides in the monterey bay area. Journal of Physical Oceanography **42**(2), 272–290 (2012)

[3] Cushman-Roisin, B., Beckers, J.-M.: Introduction to Geophysical Fluid Dynamics: Physical and Numerical Aspects. Academic press, ??? (2011)

[4] Kang, D.: Energetics and dynamics of internal tides in monterey bay using numerical simulations. Doctoral dissertation (2010)

[5] Zilberman, N., Becker, J., Merrifield, M., Carter, G.: Model estimates of m 2 internal tide generation over mid-atlantic ridge topography. Journal of Physical Oceanography **39**(10), 2635–2651 (2009)

Appendices

Appendix A Normalized topographic gradient

We calculate the normalized topographic gradient as

$$\delta h/h = \frac{\sqrt{(\partial h/\partial x)^2 + (\partial h/\partial y)^2}}{h/h_{max}} \tag{A1}$$

where h denotes the topographic depth and h_{max} is the depth of the deepest region in the domain. Thus, $\delta h/h$ is inverse proportional to the topographic depth and directly proportional to the horizontal gradient. We use this quantity as a metric to determine the regions with strong magnitudes of tidal kinetic energy production.

Appendix B Tidal parameters

The steepness parameter ϕ is defined as the ratio of the topographic gradient to the slope of the internal wave phase

$$\phi = \frac{\sqrt{(\partial h/\partial x)^2 + (\partial h/\partial y)^2}}{k/m} \tag{B2}$$

where h is the topographic depth, and $k/m = \left(\frac{\omega^2 - f^2}{N^2 - \omega^2}\right)^{1/2}$ [1] is the ratio of the horizontal to the vertical wavenumber for an internal wave with frequency ω propagating in stratified water with buoyancy frequency N^2. The steepness parameter ϕ is used to distinguish between sub-critical ($\phi < 1$), critical ($\phi = 1$), and super-critical ($\phi > 1$) topography.

Tidal excursion parameter is defined as the ratio of the length-scale covered by the barotropic tidal velocity amplitude, to the topographic length-scale. Mathematically, the excursion parameter ψ is expressed as

$$\psi = \frac{U_0}{\omega \lambda} \tag{B3}$$

where U_0 is the amplitude of the barotropic tidal velocity, ω is the tidal frequency and λ is the topographic length scale which is a representative of the horizontal dimension of the bottom mount. Following [1] and [2], we estimate the topographic length-scale as $\lambda = |h_{max} - h| / \sqrt{(\partial h/\partial x)^2 + (\partial h/\partial y)^2}$ where h_{max} is the depth of the deepest region in the domain. Thus, $|h_{max} - h|$ gives an estimate of the vertical dimension of the topographic mount from the deepest point.

Appendix C Barotropic and Baroclinic kinetic energy

The Navier-Stokes [3] prognostic equation for the velocity is expressed in tensor notation as

$$\frac{\partial u_i}{\partial t} + u_j \frac{\partial x_i}{\partial x_j} + 2\epsilon_{ijk}\sigma_j u_k = \\ -g\frac{\partial \eta}{\partial x_{i=1,2}} - \frac{1}{\rho_0}\frac{\partial p'}{\partial x_i} - \frac{g}{\rho_0}\rho'\delta_{i=3} + \frac{\partial}{\partial x_j}\left(\nu_{ij}\frac{\partial u_i}{\partial x_j}\right) \quad \text{(C4)}$$

where u_i is the velocity along the direction denoted by i, and x_j is the spatial scale along the direction j. The term $2\epsilon_{ijk}\sigma_j u_k$ denotes the Coriolis force. On the right hand side, the variable η denotes the sea-surface height, p' is the baroclinic pressure perturbation, ρ' is the density perturbation, and ν_{ij} is the eddy viscosity for the velocity u_i along direction j. The perturbation pressure is expressed as $p'(x_i,t) = p(x_i,t) - \rho_0 g(\eta - x_3) - p_0(x_3)$ where p and p_0 are the total pressure and the ambient equilibrium pressure respectively. The perturbation density is expressed as $\rho'(x_i,t) = \rho(x_i,t) - \rho_0(x_3)$, where ρ_0 is the background density.

The diffusion of velocity is expressed as $\frac{\partial}{\partial x_j}\left(\nu_{ij}\frac{\partial u_i}{\partial x_j}\right)$. At the topmost face, the vertical eddy viscosity ν_{i3} and surface wind stress $\tau_{1,2}$ (unit N/m^2) are related by the following boundary condition

$$\nu_{i,3}\frac{\partial u_i}{\partial x_3}\bigg|_{i=1,2} = -\frac{\tau_i}{\rho_0}\bigg|_{i=1,2}. \quad \text{(C5)}$$

At the bottom face, quadratic bottom drag replaces the diffusion term in the bottom boundary condition, expressed as

$$\nu_{i,3}\frac{\partial u_i}{\partial x_3}\bigg|_{i=1,2} = \frac{1}{2}c_d u_i\sqrt{u_j^2} = \frac{\kappa^2}{[ln(z/z_0)]^2}u_i\sqrt{u_j^2} \quad \text{(C6)}$$

where $c_d =$ is the bottom drag coefficient, κ is the Von-Karman constant, and z_0 is the bottom roughness length parameter.

The current velocity u_i can be expressed as $u_i = \mathbf{U}_i + \breve{u}_i$, where $\mathbf{U}_i$ and $\breve{u}_i$ are the barotropic and baroclinic components respectively. The barotropic component $\mathbf{U}_{i=1,2}$ is constant throughout the depth of the water column.

To obtain the baroclinic kinetic energy, we begin by depth-averaging the Navier-Stokes equation from the topographic depth to the surface. The depth-averaging is done in the following manner:

$$\overline{\Lambda} = \frac{\Sigma_{k=1}^{N}(\Lambda\delta z_k)}{\Sigma_{k=1}^{N}(\delta z_k)} \quad \text{(C7)}$$

where Λ is the variable at the center of each grid cell, δz_k is the vertical grid cell thickness, N is the number of grid cells along the vertical (in our case $N = 32$), and Σ denotes the summation of all vertical grid cells from 1 to N. The overline denotes the depth-average.

Multiplying equation C4 with the total velocity u_i and averaging over the entire depth, we get a prognostic equation for the depth-averaged total kinetic energy $\frac{1}{2}\overline{u_i^2}$, given as

$$\frac{1}{2}\overline{u_i^2} + \overline{u_j \frac{\partial}{\partial x_j}\left(\frac{1}{2}u_i^2\right)} = -g\frac{\partial \eta}{\partial x_i}\overline{u_i} - \frac{1}{\rho_0}\frac{\partial \overline{p' u_i}}{\partial x_i} - \frac{g}{\rho_0}\overline{\rho' u_i}\delta_{i=3} + \frac{\partial}{\partial x_j}\left[\overline{\nu_{ij}\frac{\partial}{\partial x_j}\left(\frac{1}{2}u_i^2\right)}\right] \overline{-\nu_{ij}\left(\frac{\partial u_i}{\partial x_j}\right)^2}, \quad \text{(C8)}$$

where, the overline denotes the depth average.

Now, we multiply equation C4 with the barotropic velocity component $\mathbf{U}_{i=1,2}$ to get a prognostic equation for the barotropic kinetic energy $\overline{\frac{1}{2}\mathbf{U}_i^2}$, where $i = 1, 2$ denotes only the horizontal components. The barotropic kinetic energy (E_{BTK}) prognostic equation using the tensor $i = 1, 2$ is given as

$$\frac{1}{2}\mathbf{U}_i^2 + \underbrace{\mathbf{U}_j\frac{\partial}{\partial x_j}\left(\frac{1}{2}\mathbf{U}_i^2\right)}_{ADVt} + \underbrace{\mathbf{U}_i\frac{\partial}{\partial x_j}\overline{\breve{u}_i\breve{u}_j}}_{St} = \underbrace{-g\frac{\partial \eta}{\partial x_i}\mathbf{U}_i}_{SSH} \underbrace{-\frac{1}{\rho_0}\frac{\partial \overline{p'}}{\partial x_i}\mathbf{U}_i}_{Pt} + Dt + \epsilon_t \quad \text{(C9)}$$

where, $ADVt$ is the depth-averaged advective work of E_{BTK}, and St is the depth-averaged product of the baroclinic stress divergence and the barotropic velocity. The terms Dt and ϵ_t are the depth-averaged diffusion and dissipation of E_{BTK} respectively. The depth-averaged diffusion term Dt is given as

$$Dt = \frac{\partial}{\partial x_j}\left[\overline{\nu_{ij}}\frac{\partial}{\partial x_j}\left(\frac{1}{2}\mathbf{U}_i^2\right)\right] + \mathbf{U}_i\frac{\partial}{\partial x_j}\left[\overline{\nu_{ij}\frac{\partial \breve{u}_i}{\partial x_j}}\right], \quad \text{(C10)}$$

and the dissipation ϵ_t is expressed as

$$\epsilon_t = -\overline{\nu_{ij}}\frac{\partial}{\partial x_j}\left(\frac{\partial \mathbf{U}_i}{\partial x_j}\right)^2. \quad \text{(C11)}$$

Subtracting the E_{BTK} prognostic equation C9 from the total kinetic energy equation C8, we obtain a depth-averaged prognostic equation for the baroclinic kinetic energy E_{BCK}, expressed as $\overline{\frac{1}{2}(\breve{u}_{1,2}^2 + u_3^2)}$

Thus, the equation for E_{BCK} is given as

$$\frac{\partial}{\partial t}\overline{\left(\frac{\breve{u}_{1,2}^2+u_3^2}{2}\right)}+\underbrace{\overline{\left[u_j\frac{\partial}{\partial x_j}\left(\frac{\breve{u}_{1,2}^2+u_3^2}{2}\right)\right]}}_{ADVc}+\underbrace{\overline{(\breve{u}_{1,2}\breve{u}_{2,1})}\frac{\partial \mathbf{U}_{1,2}}{\partial x_{2,1}}}_{Sc}=$$
$$\underbrace{-\frac{1}{\rho_0}\overline{\left[\breve{u}_{1,2}\frac{\partial p'}{\partial x_{1,2}}+u_3\frac{\partial p'}{\partial x_3}\right]}}_{Pc}+\underbrace{-\frac{g}{\rho_0}\overline{(\rho' u_3)}}_{Bc}+Dc+\epsilon_c \quad \text{(C12)}$$

where the terms Dc and ϵ_c denote the depth-averaged diffusion of $\overline{E_{BCK}}$ and the dissipation of E_{BCK} respectively. The baroclinic stress term $\overline{\breve{u}_{1,2}\breve{u}_{2,1}}$ is analogous to the turbulent Reynolds stress obtained by Reynolds averaging of the Navier-Stokes equation. The term Sc represents the extraction of energy from the barotropic lateral shear by the baroclinic lateral stress. Since the depth averaging is done throughout the entire water column, the eddy viscosities at the surface and bottom faces are replaced by the corresponding boundary conditions for the wind stress (equation C5) and bottom stress (equation C6) respectively. The depth-averaged diffusion of E_{BCK} is Dc, and is given as

$$Dc=\overline{\left[\frac{\partial}{\partial x_j}\left(\nu_{ij}\frac{\partial}{\partial x_j}\left(\frac{1}{2}\breve{u}_i^2\right)\right)+\left(\breve{u}_i\frac{\partial}{\partial x_j}\left(\nu_{ij}\frac{\partial \mathbf{U}_i}{\partial x_j}\right)\right)\right]}_{i=1,2}+$$
$$\frac{\partial}{\partial x_j}\overline{\left[\nu_{3j}\frac{\partial}{\partial x_j}\left(\frac{1}{2}u_3^2\right)\right]}. \quad \text{(C13)}$$

The depth-averaged dissipation of E_{BCK} is ϵ_c, and is given as

$$\epsilon_c=-\overline{\left[\nu_{ij}\left(\frac{\partial \breve{u}_i}{\partial x_j}\right)^2\right]}_{i=1,2}-\overline{\left[\nu_{3j}\left(\frac{\partial u_3}{\partial x_j}\right)^2\right]}. \quad \text{(C14)}$$

The ϵ_c is a summation of the horizontal component ϵ_h parameterized with lateral shear ($j=1,2$), and the vertical component ϵ_v parameterized using vertical shear ($j=3$).

Appendix D Energy fluxes

In the equations C9 and C12, the advective transport and pressure transport terms are expressed as divergence of the pressure and advective fluxes. From these equations, we can derive the depth-averaged horizontal energy fluxes for the barotropic and baroclinic kinetic energy due to the advection and pressure.

For the barotropic kinetic energy E_{BTK}, the fluxes due to advection (F_{ADVt}), barotropic pressure by perturbations in the sea-surface height

(F_{SSH}), and the baroclinic pressure perturbation (F_{Pt}) are given as

$$F_{ADVt} = \frac{1}{2}\mathbf{U}_j^2\mathbf{U}_i, \tag{D15}$$

$$F_{SSH} = -g\eta\mathbf{U}_i, \tag{D16}$$

and

$$F_{Pt} = -\frac{1}{\rho_0}\overline{p'}\mathbf{U}_i \tag{D17}$$

where the tensors i, j represent only the horizontal directions. All flux quantities are depth-averaged.

For the baroclinic kinetic energy E_{BCK}, the horizontal pressure energy flux (F_{Pc}) and the advective energy flux (F_{ADVc}) are given as

$$F_{Pc} = \frac{1}{\rho_0}\overline{(p'\breve{u}_i)}_{i=1,2}, \tag{D18}$$

and

$$F_{ADVc} = \overline{\left[\frac{1}{2}\left(\breve{u}_{1,2}^2 + u_3^2\right)u_i\right]}_{i=1,2}. \tag{D19}$$

Appendix E Conversion of the net barotropic to baroclinic energy

The net barotropic energy is the summation of the barotropic kinetic energy E_{BTK} and the perturbation potential energy due to perturbations in the sea-surface height. The net baroclinic energy is the summation of the baroclinic kinetic energy E_{BCK} and the available potential energy due to perturbations in the interior density surfaces. The rate of conversion from the net barotropic to net baroclinic energy, when averaged over the depth of the water column, is given as $C = \frac{1}{\rho_0}\overline{\rho'}gW$[4, 5], where W is the barotropic convergence rate, obtained by vertically integrating the equation of continuity.

$$W = -\frac{\partial}{\partial x_i}\left[(h+\eta)\mathbf{U}_i\right] \tag{E20}$$

where $\mathbf{U}_i$ is the barotropic horizontal velocity, h is the bottom depth, and η is the sea-surface height[2, 4].

Appendix F Potential Vorticity

In a hydrostatic numerical setup, the Ertel's potential vorticity q is expressed as

$$q = (f + \zeta_v)\frac{\partial B}{\partial z} - \frac{\partial v}{\partial z}\frac{\partial B}{\partial x} + \frac{\partial u}{\partial z}\frac{\partial B}{\partial y} \tag{F21}$$

where f and $\zeta_v = \partial v/\partial x - \partial u/\partial y$ are the Coriolis frequency and the relative vertical vorticity respectively, $B = -\frac{g}{\rho_0}\rho$ is the buoyancy, and u and v are the horizontal baroclinic velocities respectively.

Splitting the velocities u and v into the barotropic $(\mathbf{U}, \mathbf{V})$ and baroclinic $(\breve{u}, \breve{v})$ components, we get

$$\zeta_v = \underbrace{\left(\frac{\partial \mathbf{V}}{\partial x} - \frac{\partial \mathbf{U}}{\partial y}\right)}_{\zeta_{vt}} + \underbrace{\left(\frac{\partial \breve{v}}{\partial x} - \frac{\partial \breve{u}}{\partial y}\right)}_{\zeta_{vc}}, \tag{F22}$$

where, ζ_{vt} and ζ_{vc} are the barotropic and baroclinic relative vertical vorticities respectively. Applying the barotropic and baroclinic relative vorticity components to the potential vorticity equation F21, we get

$$q = \underbrace{f\frac{\partial B}{\partial z}}_{q_p} + \underbrace{\zeta_{vc}\frac{\partial B}{\partial z} - \frac{\partial \breve{v}}{\partial z}\frac{\partial B}{\partial x} + \frac{\partial \breve{u}}{\partial z}\frac{\partial B}{\partial y}}_{q_c} + \underbrace{\zeta_{vt}\frac{\partial B}{\partial z}}_{q_t}, \tag{F23}$$

where q_p is the component of the Ertel potential vorticity q due to the Earth's rotation (f is the Coriolis frequency), and q_c and q_t are the components of q by baroclinic and barotropic currents respectively.
